# Alternative splicing of *METTL3* explains apparently METTL3-independent m^6^A modifications in mRNA

**DOI:** 10.1371/journal.pbio.3001683

**Published:** 2022-07-19

**Authors:** Hui Xian Poh, Aashiq H. Mirza, Brian F. Pickering, Samie R. Jaffrey

**Affiliations:** Department of Pharmacology, Weill Cornell Medicine, Cornell University, New York, New York, United States of America; Yale University, UNITED STATES

## Abstract

*N*^6^-methyladenosine (m^6^A) is a highly prevalent mRNA modification that promotes degradation of transcripts encoding proteins that have roles in cell development, differentiation, and other pathways. METTL3 is the major methyltransferase that catalyzes the formation of m^6^A in mRNA. As 30% to 80% of m^6^A can remain in mRNA after METTL3 depletion by CRISPR/Cas9-based methods, other enzymes are thought to catalyze a sizable fraction of m^6^A. Here, we reexamined the source of m^6^A in the mRNA transcriptome. We characterized mouse embryonic stem cell lines that continue to have m^6^A in their mRNA after *Mettl3* knockout. We show that these cells express alternatively spliced *Mettl3* transcript isoforms that bypass the CRISPR/Cas9 mutations and produce functionally active methyltransferases. We similarly show that other reported *METTL3* knockout cell lines express altered METTL3 proteins. We find that gene dependency datasets show that most cell lines fail to proliferate after *METTL3* deletion, suggesting that reported *METTL3* knockout cell lines express altered METTL3 proteins rather than have full knockout. Finally, we reassessed METTL3’s role in synthesizing m^6^A using an exon 4 deletion of *Mettl3* and found that METTL3 is responsible for >95% of m^6^A in mRNA. Overall, these studies suggest that METTL3 is responsible for the vast majority of m^6^A in the transcriptome, and that remaining m^6^A in putative *METTL3* knockout cell lines is due to the expression of altered but functional METTL3 isoforms.

## Abstract 2

The modification m6A remains in mRNA after METTL3 depletion, suggesting that other m6A methyltransferases exist. This study investigates METTL3 knockouts, finding that they often escape knockout by expressing functional METTL3 hypomorphs, and demonstrating that METTL3 is indeed responsible for most m6A in mRNA.

## Introduction

*N*^6^-methyladenosine (m^6^A) is the most abundant internal mRNA modification [1–3], and its presence in mRNA is critical for cellular differentiation [4–6], cancer progression [7–9], and other cellular processes [10–14]. m^6^A in mRNA can regulate mRNA fates (reviewed by [15–17]), particularly by promoting mRNA degradation.

The first enzyme shown to catalyze m^6^A formation was METTL3 [18], which forms a heterodimer complex with METTL14 [19–21]. METTL3 contains the catalytic component. METTL14 was initially believed to have catalytic ability [21], but METTL14 is now known to be catalytically inactive [19,20]. Instead, METTL14 binds and positions RNA for methylation [19,20]. The METTL3-METTL14 complex is a component of a larger multiprotein “m^6^A writer complex” that mediates co-transcriptional mRNA methylation [17,22].

Although METTL3 is often described as the major m^6^A-forming enzyme in cells, the amount of m^6^A thought to be formed by METTL3 varies widely in different studies. The first study to knockout *Mettl3* showed that approximately 40% of m^6^A remained after *Mettl3* knockout in mouse embryonic stem cells (mESCs) [4]. The authors suggested that METTL14 may account for this 40% of m^6^A, based on the previous understanding that METTL14 was catalytic. However, a different group shortly thereafter reported that deletion of either *Mettl3* or *Mettl14* in mESCs leads to a loss of approximately 99% of m^6^A in mRNA [5]. These contradictory results have led to uncertainty about how much m^6^A in mRNA derives from METTL3.

*METTL3* has been knocked out in other cell lines and tissues. These results have shown that 30% to 80% of m^6^A can remain after *METTL3* knockout [23–33]. In U2OS osteosarcoma cells, approximately 60% of m^6^A remained after CRISPR-mediated knockout of *METTL3* [23,24]. In HEK293T human embryonic kidney cells, approximately 50% of m^6^A remained after CRISPR-mediated knockout of *METTL3* [25]. After Cre-conditional genomic deletion of *Mettl3* in mouse CD4+ T cells, 28% of m^6^A remained [30]. Since a nonnegligible amount of m^6^A persists after *METTL3* knockout, it has been speculated that other methyltransferases may have a major role in forming m^6^A in mRNA [4,31,33–35].

Here, we address the source of m^6^A in mRNA in reported *METTL3* knockout cells. We examined two different *Mettl3* knockout mESC lines, which both report loss of METTL3, but showed vastly different levels of residual m^6^A in mRNA. We show that *Mettl3* mutagenesis by CRISPR approaches can create alternatively spliced isoforms of *Mettl3*, resulting in an altered but catalytically active METTL3 protein. Thus, the residual m^6^A can be ascribed to a hypomorphic *METTL3* allele. We further show that other published *METTL3* mutant cell lines, which were intended to delete *METTL3*, retain m^6^A and express alternative METTL3 proteins. Furthermore, we show that *METTL3* is an essential gene in most cell lines, and thus, *METTL3* knockout cell lines that remain viable are likely to have generated alternatively spliced functional METTL3 proteins that bypass the CRISPR mutations. Lastly, we show that when a large deletion is created in *METTL3*, essentially all m^6^A is depleted in a fibroblast cell line. Overall, these studies argue that METTL3 is responsible for most m^6^A in mRNA, and that residual m^6^A after *METTL3* depletion usually reflects the generation of hypomorphic *METTL3* alleles and therefore incomplete *METTL3* knockout.

## Results

### Two mESC lines exhibit different levels of m^6^A after *Mettl3* knockout

To understand how much m^6^A in mRNA is catalyzed by METTL3, we examined 2 previously reported *Mettl3* knockout mESC lines. Two groups independently knocked out *Mettl3* in mESCs and reported markedly different levels of residual m^6^A levels in mRNA [4,5]. The first *Mettl3* knockout mESC line was described by Batista and colleagues from Howard Chang’s group, and used a CRISPR/Cas9 approach [4]. The guide RNAs were designed to introduce deletions in exon 2 of *Mettl3* and create premature termination codons [4]. The resulting mESC line, designated “exon2 *Mettl3* KO mESCs,” was found to have 40% residual m^6^A in mRNA. These authors understandably attributed the remaining m^6^A to METTL14 since, at that time, METTL14 was incorrectly shown to be a functional methyltransferase [21]. The second mESC line was described by Geula and colleagues from Jacob Hanna’s group [5]. This group used *loxP* sites surrounding exon 4 in *Mettl3* to delete the exon encoding the zinc finger domain (ZFD), an RNA recognition domain required for METTL3 methyltransferase activity [19,36]. This *Mettl3* knockout mESC line, designated “exon4 *Mettl3* KO mESCs,” exhibited <1% remaining m^6^A in mRNA. It is unclear why the exon2 *Mettl3* KO mESCs have high m^6^A levels when the exon4 *Mettl3* KO mESCs, which in principle should be the same, have virtually no remaining m^6^A.

**Fig 1:**
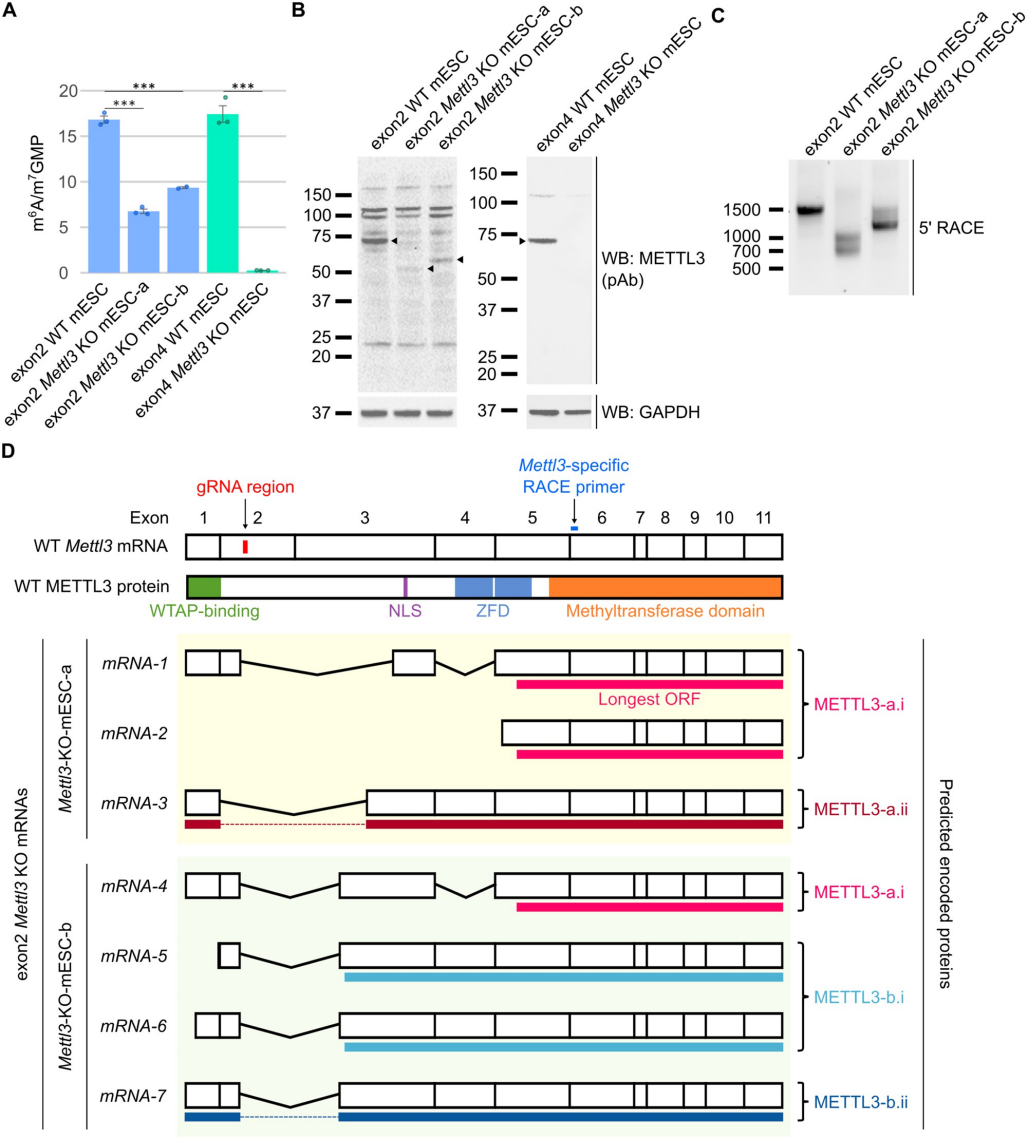
Previously described *Mettl3* KO mESC lines express shorter isoforms of *Mettl3*. (**A**) *Mettl3* KO mESCs from two groups have different m^6^A levels. To reconfirm the m^6^A levels using quantitative methods, we performed mass spectrometry to estimate the m^6^A in mRNA. Exon2 *Mettl3* KO mESCs show persistence of 40.2% (exon2 *Mettl3* KO mESC-a) and 55.6% (exon2 *Mettl3* KO mESC-b) m^6^A, respectively, while exon4 *Mettl3* KO mESCs show 1.45% m^6^A compared to WT. This confirms that exon4 *Mettl3* KO mESCs have near-complete loss of m^6^A, but not the exon2 *Mettl3* KO mESCs. Error bars indicate standard error (*n* = 3 for all, except *n* = 2 for exon2 *Mettl3* KO mESC-b). * = *p*-value < 0.5, ** = *p*-value < 0.01, *** = *p*-value < 0.005, n.s. = not significant. Underlying data can be found in S1 Data. (**B**) Exon2 *Mettl3* KO mESCs exhibit new anti-METTL3-immunoreactive bands. To investigate the effectiveness of the *Mettl3* knockout, we measured the loss of METTL3 via WB. Full-length METTL3 (75 kDa, arrowhead) was lost in both KO cell lines, but new bands, which were reactive to the anti-METTL3-antibody, appeared at approximately 50 kDa in exon2 *Mettl3* KO mESC-a and approximately 55 kDa in exon2 *Mettl3* KO mESC-b (arrowheads). This indicates the possibility that a novel smaller METTL3 protein was expressed in the exon2 *Mettl3* KO mESCs. In contrast, exon4 *Mettl3* KO mESCs have no proteins reactive to anti-METTL3-antibodies. 30 μg per lane. (**C**) 5′ RACE reveals the expression of shorter *Mettl3* mRNAs in the *Mettl3* KO mESCs. We used 5′ RACE to identify novel *Mettl3* mRNAs in the *Mettl3* KO mESCs. The full-length RACE product (approximately 1,500 bp) was lost in the *Mettl3* KO cells, but novel products at approximately 1,000 bp and approximately 700 bp were found in exon2 *Mettl3* KO mESC-a and at approximately 1,500 bp and approximately 1,300 bp in exon2 *Mettl3* KO mESC-b. These shorter mRNAs may encode the smaller METTL3 proteins seen in the KO cells. (**D**) Sequencing of 5′ RACE products show *Mettl3* mRNAs with exon skipping or alternative transcription-start sites. We sequenced the 5′ RACE products to characterize the *Mettl3* mRNA transcripts that are expressed by the exon2 *Mettl3* KO mESCs. All *Mettl3* mRNAs expressed in the KO cells skipped the guide RNA deletion region by exon skipping, or by using alternative transcription-start sites downstream of the deletion. The longest ORFs that are in-frame with the WT *Mettl3* mRNAs are shown as solid lines below each mRNA. The encoded protein is also represented, with the domains required for METTL3 activity shown. m^6^A, *N*^6^-methyladenosine; mESC, mouse embryonic stem cell; NLS, nuclear localization signal; ORF, open reading frame; pAb, polyclonal antibody; RACE, rapid amplification of cDNA ends; WB, western blot; WT, wild-type; ZFD, zinc finger domain.

We first reconfirmed the levels of m^6^A in these two cell lines. The m^6^A levels in the two *Mettl3* knockout mESC lines were originally measured via different methods, which may have led to these contradictory results. Thin-layer chromatography was used by Batista and colleagues [4], while mass spectrometry was used by Geula and colleagues [5]. We measured m^6^A in the mESC lines using mass spectrometry [37]. Our mass spectrometry measurements were consistent with the results originally reported by the two groups. In the two exon2 *Mettl3* KO mESC lines, designated exon2 *Mettl3* KO mESC-a and exon2 *Mettl3* KO mESC-b, we saw 40.2% and 55.6% residual m^6^A (Fig 1A), comparable to approximately 40% originally reported by this group [4]. However, the exon4 *Mettl3* KO mESCs had only 1.45% residual m^6^A (Fig 1A), corroborating the 0.5% remaining m^6^A originally reported by this group [5].

The near-complete loss of m^6^A in the exon4 *Mettl3* KO mESCs suggests that METTL3 is the major m^6^A writer in this mESC line. On the other hand, the exon2 *Mettl3* KO mESCs still retain m^6^A despite *Mettl3* depletion. Although it is possible that these mESCs use an alternate enzyme for m^6^A biosynthesis, we suspected that *Mettl3* was not completely knocked out in these cell lines.

### *Mettl3* knockout mESCs that retain m^6^A express alternative *Mettl3* isoforms

We next wanted to confirm that *Mettl3* was knocked out in the exon2 *Mettl3* KO mESCs. Previously, a western blot was used to determine the loss of METTL3 protein [4]. To first confirm that the METTL3 protein is indeed absent, we performed a western blot using a METTL3 polyclonal antibody raised against amino acids 229–580 of METTL3, which correspond to amino acids encoded by exons 3–11. Full-length METTL3 (approximately 75 kDa) was identified in the wild-type (WT) mESCs, and was lost in both *Mettl3* KO cell lines (Fig 1B). However, we observed new bands in the anti-METTL3 immunoblot in the knockout cell lines. The new proteins were approximately 50 kDa in exon2 *Mettl3* KO mESC-a and approximately 55 kDa in exon2 *Mettl3* KO mESC-b (Fig 1B). While nonspecific background bands are visible in all 3 cell lines, these particular proteins were not visible in the WT cell line, suggesting they are unique to the knockout cell lines and not just background. These proteins were also not visible in the exon4 *Mettl3* KO cell lines (Figs 1B and S1B).

To confirm that these are indeed METTL3 proteins, we repeated the western blot with a second anti-METTL3 antibody raised against amino acids surrounding Leu297 of METTL3, which correspond to amino acids encoded by exon 4. Again, we found the same bands in the exon2 *Mettl3* KO cell lines (S1A and S1C Fig). These proteins may have escaped notice in the original study as the study used a different antibody, which may have been unable to detect these isoforms [4]. Overall, the new METTL3-antibody-reactive proteins suggests that smaller METTL3 isoforms are produced in the exon2 *Mettl3* KO cells that may be the source of m^6^A in these cells. Although expression levels of these proteins appear low, previous studies have suggested that METTL3 is not rate limiting, and therefore, low METTL3 expression can still lead to high m^6^A levels [22].

We wanted to understand the mechanism of METTL3 expression in the exon2 *Mettl3* KO mESCs. To do this, we first determined the sequence of these isoforms. Since the exon2 *Mettl3* KO cells were produced using guide RNAs targeting exon 2 of *Mettl3* [4], any CRISPR/Cas9-induced mutations and potential alternative splicing events are likely to be in the 5′ end of the transcript. We therefore used 5′ RACE (rapid amplification of cDNA ends) [38] to identify new transcription-start sites or possible exon skipping events of the *Mettl3* transcripts expressed in these cells.

Using 5′ RACE, a single major approximately 1,500 bp band was seen for *Mettl3* in WT mESCs (Fig 1C). In contrast, in the two exon2 *Mettl3* KO cell lines, we found shorter bands indicative of *Mettl3* mRNAs with a shorter 5′ region (Fig 1C). We sequenced these 5′ RACE products and found several *Mettl3* mRNAs from the exon2 *Mettl3* KO cells (Fig 1D and S1 Table). In all cases, the mRNAs show alternative splicing that bypasses the CRISPR deletion in exon 2 by exon skipping, or use of an alternative transcription-start site downstream of the deletion.

We next asked if these mRNAs could potentially encode the altered METTL3 proteins detected in the *Mettl3* KO mESCs. For each mRNA, we identified the longest possible open reading frame (ORF) that is in frame with the METTL3 catalytic domain (Fig 1D and S2 Table). In the case of *Mettl3* KO mESC-a, we found a transcript that is predicted to encode a METTL3 protein (designated “METTL3-a.ii”) that matches the size of the altered METTL3 protein from this cell line (Fig 1B and 1D). Similarly, we identified a transcript that is predicted to encode a protein with a similar size to the altered METTL3 protein in *Mettl3* KO mESC-b (designated “METTL3-b.ii”) (Fig 1B and 1D).

Together, these data identify potential transcripts that may encode the altered METTL3 proteins found in the exon2 *Mettl3* KO cells.

### Altered METTL3 proteins expressed in incomplete *Mettl3* knockout mESCs can catalyze the formation of m^6^A in cells

**Fig 2:**
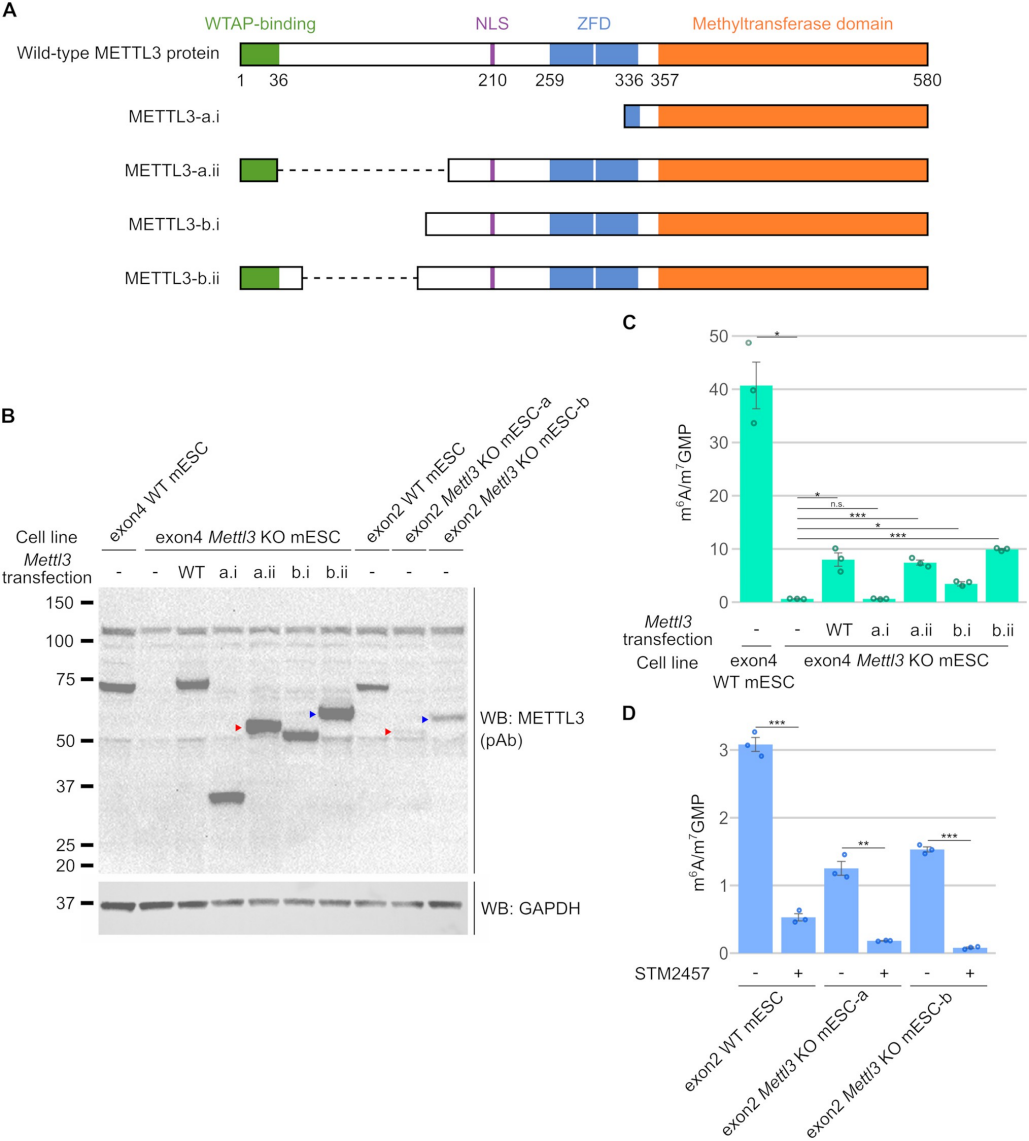
Shortened isoforms of METTL3 catalyze m^6^A formation in *Mettl3* KO mESCs. (**A**) The predicted domain structure of proteins encoded by the altered METTL3 ORFs expressed in exon2 *Mettl3* KO mESCs suggests they may be functional. The domains that are known to be necessary for m^6^A formation by METTL3 include the WTAP-binding domain [39], ZFD [19,36], and methyltransferase domain [19,20]. To determine if the METTL3 ORFs from exon2 *Mettl3* KO mESCs encode functional METTL3 proteins, we predicted the domain structure of the METTL3 protein isoforms from their ORFs. While all the predicted proteins have the methyltransferase domain, only METTL3-a.ii and METTL3-b.ii have all the known critical domains for m^6^A-writing. (**B**) WB of transfected FLAG-tagged METTL3 isoform ORFs. To determine if the METTL3 ORFs we found in the knockout cells can synthesize m^6^A, we expressed the METTL3 ORFs in exon4 *Mettl3* KO mESCs that exhibit no METTL3 protein and essentially no baseline m^6^A signal. After 48 h, the alternatively spliced METTL3 proteins can be detected by immunoblotting with an anti-METTL3 antibody. FLAG-METTL3-a.ii (50 kDa, red arrowhead) and FLAG-METTL3-b.ii (55 kDa, blue arrowhead) have similar sizes to the anti-METTL3-antibody-reactive protein seen in exon2 *Mettl3* KO mESC-a (red arrowhead) and exon2 *Mettl3* KO mESC-b (blue arrowhead), respectively. 30 μg per lane. (**C**) Isoforms of METTL3 proteins can write m^6^A. After 48 h of transfection, RNA from each sample was processed, and m^6^A was measured using mass spectrometry. Expression of full-length WT METTL3 was able to rescue 19.7% of the m^6^A. METTL3-a.i was unable to rescue m^6^A, but METTL3-a.ii was able to rescue 18.3% of m^6^A. METTL3-b.i could only rescue 8.5% of m^6^A, whereas METTL3-b.ii rescued 24.4% of m^6^A, respectively. Thus, METTL3-a.ii and METTL3-b.ii proteins that are expressed in the exon2 *Mettl3* KO mESCs are able to catalyze the formation of m^6^A. Error bars indicate standard error (*n* = 3). * = *p*-value < 0.5, ** = *p*-value < 0.01, *** = *p*-value < 0.005, n.s. = not significant. Underlying data can be found in S1 Data. (**D**) A METTL3-specific inhibitor leads to loss of m^6^A even in exon2 *Mettl3* KO mESCs. Exon2 WT and *Mettl3* KO mESCs were treated with 30 μM STM2457, a METTL3-specific inhibitor, and m^6^A levels were measured by mass spectrometry after 48 h. STM2457 treatment reduced m^6^A by 82.8% in the WT mESCs. In exon2 *Mettl3* KO mESC, m^6^A was reduced by 85.4% in *Mettl3* KO mESC-a after STM2457 treatment and by 94.8% in *Mettl3* KO mESC-b. Thus, METTL3 is responsible for the remaining m^6^A in the exon2 *Mettl3* KO mESCs. Residual m^6^A after STM2457 treatment may reflect incomplete inhibition of METTL3 at 30 μM. Error bars indicate standard error (*n* = 3). * = *p*-value < 0.5, ** = *p*-value < 0.01, *** = *p*-value < 0.005, n.s. = not significant. Underlying data can be found in S1 Data. m^6^A, *N*^6^-methyladenosine; mESC, mouse embryonic stem cell; NLS, nuclear localization signal; ORF, open reading frame; pAb, polyclonal antibody; WB, western blot; WT, wild-type; ZFD, zinc finger domain.

We next asked if the novel *Mettl3* transcript isoforms encode functional m^6^A-forming methyltransferases. METTL3 contains several domains necessary for methyltransferase activity, including the WTAP-binding domain [39], the ZFD [19,36], and the methyltransferase domain [19,20]. Both METTL3-a.ii and METTL3-b.ii contain all 3 domains, and therefore may be functional. Other altered *Mettl3* transcripts in the *Mettl3* KO mESCs encode predicted proteins (designated “METTL3-a.i” and “METTL3-b.i”) that lack one or more of these domains (Fig 2A). Notably, the methyltransferase domain is required for interaction with METTL14 [20,39], and METTL3 stabilizes METTL14 through this interaction [6,19]. The exon4 *Mettl3* KO mESCs were shown to have complete loss of METTL14 protein, supporting that METTL3 is required for METTL14 stability [5]. On the other hand, the exon2 *Mettl3* KO mESCs were shown to continue to express METTL14 [4], again suggesting that they express a METTL3 protein that was able to interact with and stabilize METTL14.

To test the activity of these METTL3 isoforms, we expressed FLAG-tagged METTL3 isoform constructs in the exon4 *Mettl3* KO mESCs, which lack METTL3-immunoreactive bands (Figs 1B, S1B and S1C) and lack m^6^A in mRNA (Fig 1A). First, we noted that the size of METTL3-a.ii and METTL3-b.ii were indeed similar to the sizes of the smaller METTL3 proteins in *Mettl3* KO mESC-a and *Mettl3* KO mESC-b, respectively (Fig 2B). Thus, METTL3-a.ii and METTL3-b.ii may be the novel METTL3 isoforms we detected in the exon2 *Mettl3* KO mESCs. In contrast, bands corresponding to METTL3-a.i (approximately 30.4 kDa) and METTL3-b.i (approximately 49 kDa) were not seen in *Mettl3* KO mESC-a or *Mettl3* KO mESC-b, respectively (Figs 1B and 2B). These METTL3 isoforms may have been less efficiently transcribed or translated, or may be less stable, leading to the lack of an immunoreactive band for these proteins.

We first tested expression of full-length METTL3 in the exon4 *Mettl3* KO mESCs and found that they rescued m^6^A levels to 19.7% of WT (Fig 2C). The lack of complete rescue by the WT *Mettl3* transcript may be due to inefficient or uneven transfection of the cell population (S2 Fig). METTL3-a.ii and METTL3-b.ii were able to rescue 18.3% and 24.4% of the m^6^A, respectively (Fig 2C), suggesting that they are functional m^6^A methyltransferases. As predicted, METTL3-a.i failed to rescue m^6^A and METTL3-b.i could only rescue a small portion of m^6^A (8.5%) (Fig 2C). Overall, these data indicate that the exon2 *Mettl3* KO mESCs express hypomorphic *Mettl3* alleles that encode catalytically active METTL3 isoforms, METTL3-a.ii and METTL3-b.ii. Thus, the exon2 *Mettl3* KO mESCs should be viewed as incomplete knockouts with hypomorphic *Mettl3* alleles.

We wanted to find out if the mRNAs encoding METTL3-a.ii and METTL3-b.ii were translated in the cells to produce their respective proteins. To test this, we performed polysome profiling on the exon2 *Mettl3* KO mESCs. We collected fractions corresponding to sub-monosomal, light polysome, medium polysome, and heavy polysome fractions (S3 Fig). Using PCR primers, we amplified *Mettl3* transcript isoforms in each fraction. We found *Mettl3* PCR amplification products in the heavy polysome fraction (S3 Fig). Sequencing confirmed that these PCR products are *mRNA-3* that encodes METTL3-a.ii in *Mettl3* KO-mESC-a and *mRNA-7* that encodes METTL3-b.ii in *Mettl3* KO mESC-b (S3 Fig and S3 Table). Thus, these transcripts are indeed translated.

We also used an orthogonal pharmacological approach to determine if a METTL3 isoform could be responsible for the m^6^A produced in the exon2 *Mettl3* KO mESCs. STM2457 is a METTL3 inhibitor that was developed by a structure-based approach to be highly specific to METTL3 [40]. Screens with other methyltransferases showed that STM2457 does not affect the activity of other known RNA methyltransferases [40]. Thus, we treated the exon2 *Mettl3* KO cells with STM2457 to investigate if the remaining m^6^A in these cells was produced by METTL3. STM2457 treatment reduced m^6^A levels in the WT mESCs by 82.8% after 48 h (Fig 2D). Similarly, we saw that STM2457 treatment reduced m^6^A by 85.4% in exon2 *Mettl3* KO mESC-a and 94.8% in exon2 *Mettl3* KO mESC-b (Fig 2D), indicating that this m^6^A was produced by METTL3. The small amount of remaining m^6^A could be either due to incomplete inhibition by STM2457, or a small amount of m^6^A catalyzed by a different methyltransferase. On the other hand, STM2457 treatment had no effect on m^6^A levels in the exon4 *Mettl3* KO mESCs (S4 Fig). This suggests that METTL3 is indeed responsible for the remaining m^6^A in the exon2 *Mettl3* KO mESCs.

### *METTL3* knockout U2OS cells express an altered METTL3 protein

The idea that METTL3 is not responsible for all m^6^A in mRNA was further supported by *METTL3* knockout in several cell lines, each of which show high residual levels of m^6^A [23–29]. For example, a *METTL3* KO U2OS cell line was reported to have approximately 60% of the m^6^A remaining compared to WT [23,24]. Similarly, we found that a previously reported *METTL3* KO A549 cell line [41] has approximately 90% of m^6^A remaining compared to the WT (S5 Fig). The persistence of m^6^A in these *METTL3* knockout cell lines has led to the idea that other enzymes mediate a substantial fraction of m^6^A in mRNA. However, in light of our finding that alternatively spliced *Mettl3* isoforms were induced in the *Mettl3* knockout mESCs, another possibility is that *METTL3* isoforms were induced in these *METTL3* knockout cell lines as well, which led to the high m^6^A levels in these *METTL3* knockout cell lines.

**Fig 3:**
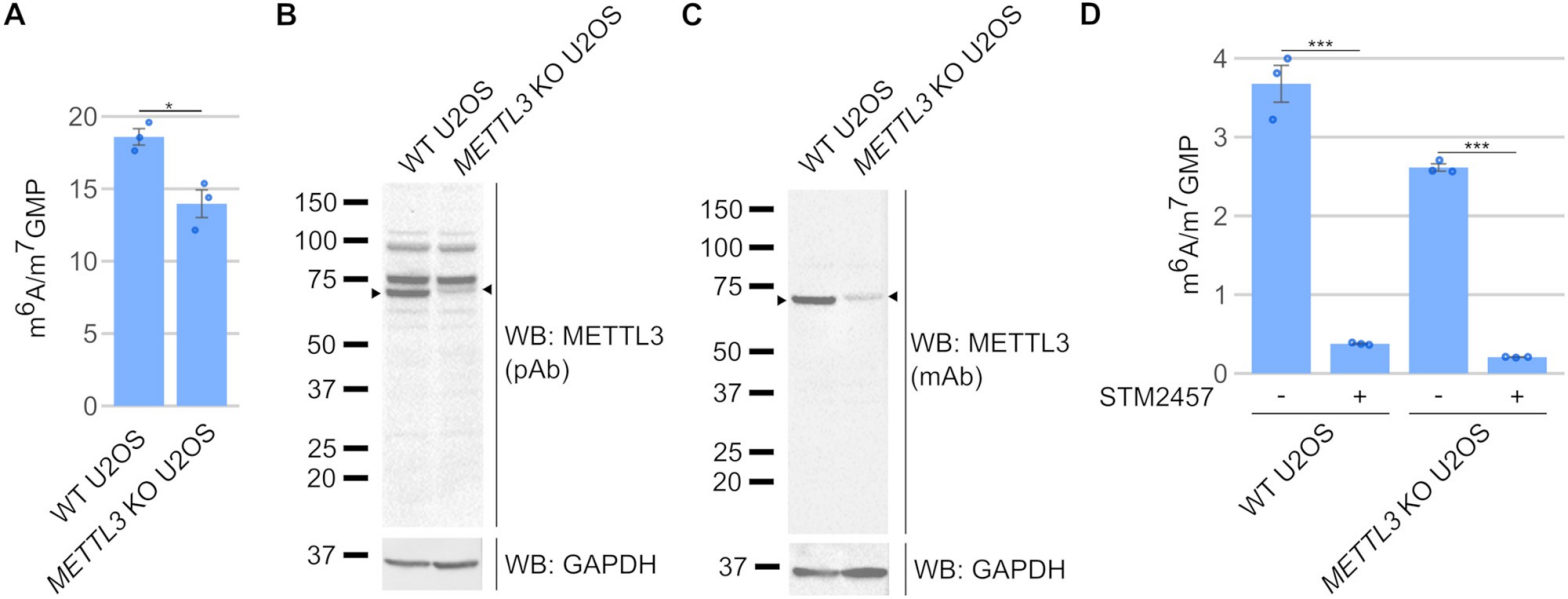
*METTL3* knockout in U2OS cells also appears to be incomplete. (**A**) *METTL3* KO U2OS cells have persistent m^6^A. *METTL3* KO U2OS cells have been reported to have 60% the levels of m^6^A found in control U2OS cells [24]. We reconfirmed this with mass spectrometry measurements of m^6^A, which showed that *METTL3* KO U2OS cells have 75.2% remaining m^6^A compared to WT. Thus, m^6^A levels remain high in *METTL3* KO U2OS cells. Error bars indicate standard error (*n* = 3). * = *p*-value < 0.5, ** = *p*-value < 0.01, *** = *p*-value < 0.005, n.s. = not significant. Underlying data can be found in S1 Data. (**B**) *METTL3* KO U2OS cells express a novel anti-METTL3-antibody-reactive protein. As m^6^A levels were not completely ablated in the *METTL3* KO U2OS cells, we assessed METTL3 protein expression in these cells to confirm if the knockout was effective. We found that WT METTL3 protein was lost in the *METTL3* KO U2OS cells, but a larger protein that was reactive to the anti-METTL3 antibody was found in the *METTL3* KO U2OS cells. This suggests the *METTL3* KO U2OS cells express a novel METTL3 protein that is slightly larger than the WT METTL3. (**C**) Confirmation of a novel METTL3-like protein in *METTL3* KO U2OS using a second METTL3 antibody. To confirm that the METTL3-immunoreactive band we saw in *METTL3* KO U2OS cells in Fig 3B was METTL3, we used a second anti-METTL3 mAb to confirm the result. The same protein band is immunoreactive to the second anti-METTL3 antibody, thus suggesting that the *METTL3* KO U2OS cells express a novel, larger METTL3 protein. (**D**) A METTL3-specific inhibitor leads to loss of m^6^A even in *METTL3* KO U2OS cells. WT and *METTL3* KO U2OS cells were treated with 30 μM STM2457, and m^6^A levels were measured by mass spectrometry after 48 h. m^6^A was reduced by 89.8% in the WT U2OS cells and 92.1% in the *METTL3* KO U2OS cells after STM2457 treatment. It should be noted that 30 μM may not fully inhibit METTL3, so some of the residual m^6^A after STM2457 treatment may still derive from METTL3 isoforms. Thus, a METTL3 isoform is responsible for most of the remaining m^6^A in the *METTL3* KO U2OS cells. Error bars indicate standard error (*n* = 3). * = *p*-value < 0.5, ** = *p*-value < 0.01, *** = *p*-value < 0.005, n.s. = not significant. Underlying data can be found in S1 Data. mAb, monoclonal antibody; m^6^A, *N*^6^-methyladenosine; pAb, polyclonal antibody; WB, western blot; WT, wild-type.

We examined one of these cell lines—the *METTL3* knockout U2OS cell line that was generated using CRISPR/Cas9-directed mutagenesis with a guide RNA targeting exon 1 [23]. We first reconfirmed the m^6^A levels in these *METTL3* knockout cells and found that the *METTL3* knockout U2OS cells retained 75.2% m^6^A compared to the WT (Fig 3A), comparable to the 60% originally reported [24]. Hence, *METTL3* knockout U2OS cells continue to have high levels of m^6^A in mRNA.

We next asked if the *METTL3* KO U2OS cells express a METTL3 protein using a western blot. We observed full-length METTL3 in the WT U2OS cells, but not in the knockout cells (Fig 3B). However, a slightly larger anti-METTL3 immunoreactive band was detected exclusively in the knockout cells (Fig 3B). To confirm that this is indeed a METTL3 isoform, we validated the western blot with a second anti-METTL3 monoclonal antibody and found that the protein in the knockout cells was also reactive to the second anti-METTL3 antibody (Fig 3C). We note that we could observe a similar protein band in the authors’ original western blot that used a different METTL3 antibody [23]; however, it was much fainter and could easily be mistaken for nonspecific background. Thus, this suggests that the *METTL3* KO U2OS cells continued to express a METTL3 protein.

We wanted to find out if METTL3 could be responsible for the m^6^A produced in the *METTL3* KO U2OS cells using the METTL3-specific inhibitor, STM2457 [40]. STM2457 treatment reduced m^6^A levels in the WT U2OS cells by 89.8% after 48 h (Fig 3D). Similarly, STM2457 treatment reduced m^6^A levels by 92.1% in the *METTL3* KO U2OS cells (Fig 3D). These data suggest that a METTL3 isoform is responsible for most of the remaining m^6^A in the *METTL3* KO U2OS cells. Furthermore, the depletion of approximately 90% m^6^A by STM2457 further suggests that METTL3 is responsible for the majority of m^6^A in U2OS cells. Overall, this data again suggests that CRISPR/Cas9 mutagenesis results in the appearance of a novel METTL3 isoform in a *METTL3* knockout cell line, which could explain m^6^A persistence in these cells.

### *METTL3* knockout cell lines are generally not viable

Several *METTL3* knockout cell lines have been reported despite the fact that *METTL3* is thought to be an essential gene. *Mettl3* knockout is embryonic lethal in mice at E5.5 before cell specification occurs [5], and CRISPR screens have indicated that *METTL3* is an essential gene in specific cell lines that were tested [7,42]. On the other hand, the exon4 *Mettl3* knockout mESCs are able to survive, demonstrating that some cell lines can survive without *METTL3* or m^6^A. Therefore, it is not clear which cell lines require *METTL3* for survival. If *METTL3* is required for survival of most cell lines, it would cast doubt on the stable *METTL3* knockout cell lines that have been reported in the literature.

**Fig 4:**
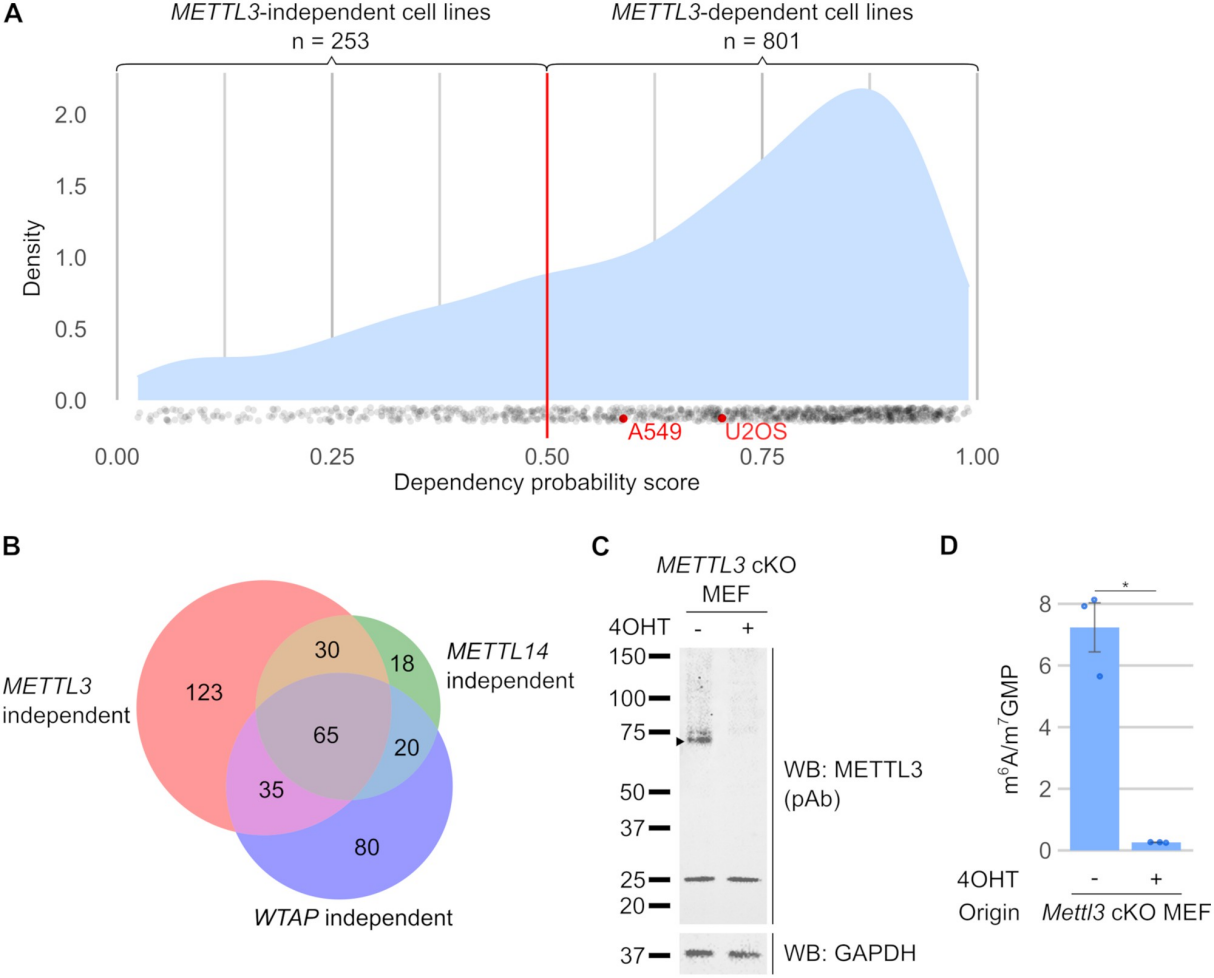
Conditional *METTL3* knockouts can be used to study m^6^A when stable *METTL3* knockouts are not viable. (**A**) Most cell lines are dependent on *METTL3* for growth. Mouse studies previously indicated that *Mettl3* is an essential gene for early embryonic survival [5], so we wanted to know which cell lines are dependent on *METTL3*. Using the CRISPR gene dependency probability score from the DepMap 21Q4 dataset [43–47], we found that 801 of 1,054 cell lines are dependent on *METTL3* (dependency probability score >0.5). Therefore, most cells lines will not survive after *METTL3* knockout. The density plot shows the overall distribution of dependency probability scores, while each individual cell line is represented as a dot. U2OS and A549 cell lines (red), where *METTL3* has been previously knocked out, are shown here to be dependent on *METTL3* and thus should not be viable after *METTL3* knockout. Underlying data for this figure was extracted from the DepMap 21Q4 dataset [47]. (**B**) A small subset of cell lines may be m^6^A independent. Although most cell lines are dependent on *METTL3*, a small subset of cell lines can survive despite *METTL3* knockout. To identify cell lines that we can confidently consider m^6^A-independent, we obtained a list of cell lines whose survival is also independent of other members of the m^6^A writer complex, *METTL3*, *METTL14*, and *WTAP*. This approach suggests that 65 cell lines may be able to proliferate in an m^6^A-independent manner (S4 Table). Underlying data for this figure was extracted from the DepMap 21Q4 dataset [47]. (**C**) *Mettl3* conditional knockout MEFs do not express METTL3 protein. To determine the amount of m^6^A in mRNA that can be attributed to *Mettl3*, we generated a tamoxifen-inducible *Mettl3* conditional knockout MEF cell line. We used WB to validate the loss of METTL3. After 5 days of 4-hydroxytamoxifen treatment (500 nM), we observed loss of the WT METTL3 protein. 30 μg per lane. (**D**) *Mettl3* conditional knockout MEFs show near-complete loss of m^6^A. We measured m^6^A levels in mRNA derived from tamoxifen-inducible *Mettl3* conditional knockout MEFs. Eight days after 4OHT treatment (500 nM), the *Mettl3* KO MEFs showed 3.6% remaining m^6^A. Hence, m^6^A is almost completely lost after *Mettl3* knockout in MEFs. Error bars indicate standard error (*n* = 3). * = *p*-value < 0.5, ** = *p*-value < 0.01, *** = *p*-value < 0.005, n.s. = not significant. Underlying data can be found in S1 Data. m^6^A, *N*^6^-methyladenosine; MEF, mouse embryonic fibroblast; pAb, polyclonal antibody; WB, western blot; 4OHT, 4-hydroxytamoxifen.

To understand which cell lines require *METTL3* for survival, we screened the Cancer Dependency Map Project (DepMap) 21Q4 dataset [43–47]. This dataset measures cell proliferation in 1,054 cell lines following a CRISPR loss-of-function screen. Failure of a cell line to grow after expression of the guide RNA that inactivates a gene indicates that the gene is essential in the cell line. The probability that a cell line is dependent on each gene is calculated as a gene dependency probability score, which accounts for guide efficacy as well as copy number of each gene. The 21Q4 DepMap dataset [47] showed that *METTL3* is necessary for cell proliferation in 801 of 1,054 tested cell lines (Fig 4A). To account for the possibility of off-target effects in the DepMap CRISPR screen, we further looked for *METTL3*-dependent cell lines that were also dependent on other members of the writer complex, *METTL14* and *WTAP* [6,21,22,48]. We found that of the 801 cell lines dependent on *METTL3*, 683 were also dependent on *METTL14* and *WTAP* (S6 Fig), suggesting that these cell lines indeed require the m^6^A methyltransferase complex for proliferation. The *METTL3*-dependent cell lines include the U2OS and A549 cell lines that other groups have used to make *METTL3* knockout cell lines [23,41] (Fig 4A). The DepMap data suggests that these reported *METTL3* cell lines should not have been viable.

It is important to note that the DepMap dataset measures proliferation after a CRISPR loss-of-function screen. Even if a small number of cells manage to escape the CRISPR inactivation of *METTL3* and continue to proliferate, the overall reduction in proliferation will still be reflected in the DepMap gene dependency score. On the other hand, during generation of *METTL3* knockout cell lines, researchers are actively selecting for cells that continue to proliferate, therefore potentially selecting for cells that have escaped the knockout.

Thus, this leads us to believe that reported stable *METTL3* knockout cell lines were able to survive because they were selected for their ability to produce a functional alternatively spliced *METTL3* isoform that bypasses the CRISPR mutation, similar to the exon2 *Mettl3* KO mESCs. This alternative *METTL3* isoform also explains why these cells retain high levels of m^6^A. Thus, the reported *METTL3* knockout cell lines are likely not true *METTL3* knockouts.

However, a few cell lines are able to survive after *METTL3* knockout. The exon4 *Mettl3* KO mESCs are one such example of a cell that can survive without METTL3 [5]. A group has also reported that mouse CD4+ T cells can survive after *Mettl3* deletion [30]. The 21Q4 DepMap dataset shows that only 253 cell lines are not dependent on *METTL3*. Furthermore, only 65 cell lines were not dependent on any of the members of the m^6^A writer complex, which includes *METTL3*, *METTL14*, and *WTAP* [6,21,22,48] (Fig 4B and S4 Table). This small subset of cell lines may be the best cell lines for creating *METTL3* knockout cells, since loss of m^6^A in the other cell lines will most likely lead to cell death unless an alternatively spliced METTL3 isoform is selected for. Thus, any reported stable *METTL3* knockout in cell lines other this small subset of m^6^A-independent cell lines should be more carefully evaluated.

### METTL3 is responsible for all m^6^A in mRNA in mouse embryonic fibroblasts

Although the full *Mettl3* knockout in mESCs led to near complete loss of m^6^A [5], this may be a unique feature of embryonic stem cells. It is possible that other cell types can compensate for METTL3 depletion using other methyltransferases that can synthesize m^6^A. To find out if METTL3 is the major writer of m^6^A in mRNA in cell lines other than mESCs, we needed to deplete METTL3 in a different cell line, and measure the remaining amount of m^6^A. Because stable *METTL3* knockout cells are generally not viable, we produced *Mettl3* conditional knockout mouse embryonic fibroblast (MEF) cell lines derived from *Mettl3*^*flox/flox*^ mice. *loxP* sites flanking exon 4 of METTL3 were inserted in the mouse genome [32]. Upon tamoxifen-induced expression of Cre, recombination of the *loxP* sites leads to deletion of exon 4 that encodes the ZFD, similar to how *Mettl3* is deleted in the exon4 *Mettl3* KO mESCs [5].

Using the tamoxifen-inducible *Mettl3* knockout MEFs, we found that METTL3 protein expression was completely lost 5 days after tamoxifen treatment (Figs 4C and S7A). By day 6, we observed that MEF proliferation was markedly reduced (S7B Fig), corroborating that *METTL3* is essential for proliferation, and therefore stable *METTL3* knockout cell lines are unlikely to survive.

We next determined the fraction of m^6^A in mRNA that is catalyzed by METTL3 in MEFs. We found that 8 days after tamoxifen treatment, most of the m^6^A was lost, with only 3.6% of m^6^A remaining (Fig 4D). Thus, this demonstrates that aside from mESCs, METTL3 is also the predominant m^6^A methyltransferase in MEFs.

## Discussion

Here, we address the source of METTL3-independent m^6^A, which is widely discussed in the literature and have been described based on high levels of residual m^6^A after knockdown or knockout of *METTL3* [4,6–8,21–32,41,42,48–52]. This has led to the idea that some m^6^A is catalyzed by METTL3, while another substantial fraction of m^6^A is catalyzed by other enzymes. Our results show that the residual m^6^A seen after *METTL3* knockout can be readily explained by the induction of alternatively spliced hypomorphic *METTL3* alleles and the subsequent expression of altered METTL3 proteins. We show that a widely used “*Mettl3* knockout” mESC line [4] undergoes alternative splicing to bypass the CRISPR/Cas9-induced mutations, creating a smaller but catalytically active METTL3 protein. We further show that another published *METTL3* knockout U2OS cell line that also has been shown to contain high levels of residual m^6^A [23,24] also exhibits an altered METTL3 protein. These studies show that altered METTL3 proteins likely account for the residual m^6^A in the cells, rather than an alternative methyltransferase. The evidence for the importance of METTL3 alone as the major m^6^A-forming enzyme is supported by our data in which deleting *METTL3* in MEFs using a large exon 4 deletion leads to a loss of >95% of m^6^A, and by our data where METTL3 inhibition by STM2457 lead to 90% loss of m^6^A in U2OS cells. Lastly, we demonstrate that METTL3 is essential for the proliferation and growth of the vast majority of cell lines and therefore, many reported stable *METTL3* knockout cell lines are unlikely to be true knockout cells since they would not be able to proliferate, with a few exceptions. Overall, our studies resolve an important long-standing inconsistency in the literature and argue that (1) METTL3 is the major source of m^6^A in mRNA; and (2) m^6^A that remains after *METTL3* knockout is likely due to cells escaping *METTL3* deletion by creation of new *METTL3* isoforms.

Our data suggests that *METTL3* knockout can be validated by measuring residual m^6^A levels in mRNA. We have shown here that several cell lines described to be *METTL3* knockouts still express METTL3 isoforms and have high levels of m^6^A [23–29]. *METTL3* knockout cells that continue to have m^6^A should not be used to determine the function of m^6^A in any biological process. Because these cells retain considerable amounts of m^6^A, they cannot be used to identify pathways and processes that require m^6^A. Furthermore, these cells may express isoforms of METTL3 with unknown functions and properties. These nonphysiological METTL3 isoforms may lead to unexpected results, which may be conflated with findings that arise from loss of m^6^A. We note that we observed multiple unique METTL3 isoforms in the different cell lines we characterized, all of which are not visible in the WT cells. These METTL3 isoforms are likely to have been generated by stochastic alternative splicing events, which were eventually selected for as the production of a functional METTL3 gave the cell a proliferative advantage. Thus, each CRISPR knockout cell line may produce different METTL3 isoforms, each with unique unpredictable properties.

To more reliably inactivate *METTL3*, genomic regions encoding critical enzymatic domains should be deleted, rather than simply mutated, since small mutations may be more readily bypassed through alternative splicing events. Furthermore, as *METTL3* knockout is not viable in most cell lines, conditional *METTL3* knockouts provide a useful model for studying m^6^A. METTL3 deletion and m^6^A levels must be carefully documented before reporting these cell lines as *METTL3* knockouts. m^6^A quantification will also allow a better understanding if partial or complete losses of m^6^A are able to mediate the studied outcomes.

Although our results indicate that METTL3 accounts for the vast majority (>95%) of m^6^A in mRNA in MEFs and mESCs, numerous studies have shown that *METTL3* knockout results in a large amount of residual m^6^A in mRNA. Three major technical problems likely account for this “METTL3-independent” m^6^A:

1. Hypomorphic *METTL3* alleles. In this study, we characterized a variety of diverse alternative splicing events that occur in “*Mettl3* knockout” mESCs. In these cells, *Mettl3* was targeted using CRISPR/Cas9 systems to create indels and frameshifts. Although this is a reasonable method for gene inactivation [53,54] and may have initially inactivated *Mettl3*, cells which develop alternative splicing patterns that enable formation of an active METTL3 enzyme will gain a proliferative advantage. It is likely that after *METTL3* knockout, cells in which *METTL3* was truly inactivated will stop proliferating. At the same time, any cells that can express a *METTL3* isoform that skips the mutation will gain a proliferative advantage, and will be further selected for their ability to up-regulate this isoform. These escaped cells will then be expanded and incorrectly reported as a “*METTL3* knockout” cell line. Similar alternative splicing events that bypass CRISPR/Cas9-induced mutations have been reported in studies of inactivation of other genes [55–57]. Alternative splicing may allow bypass of CRISPR/Cas9-mediated *METTL3* knockout in other cell lines.
2. Heterogeneity of cell population. In a population of *METTL3* knockout cells, any contaminating cells that express functional METTL3 will lead to some level of m^6^A being produced and detected. For example, mouse studies frequently use tissue-specific knockout systems to deplete *Mettl3* in a specific tissue as *Mettl3* knockout is embryonic lethal [5,30–32]. When isolating these tissues, contamination from other cells in the tissue, such as endothelial or immune cells, could lead to detectable m^6^A levels in the samples. Additionally, Cre expression is variable, and can lead to a lack of knockout in some cells [58]. Thus, residual m^6^A in these experiments may simply reflect a mixed population of knockout and non-knockout cells. METTL3 levels can be measured by immunofluorescence to determine if knockout is heterogeneous in these populations.
3. Misattribution of background noise in m^6^A mapping studies as m^6^A sites. A common approach for studying m^6^A sites is MeRIP-seq [3,59], and other antibody-based sequencing methods to detect m^6^A across the transcriptome [60]. In numerous studies, researchers knocked down or deleted *METTL3* and regarded the remaining m^6^A “peaks” as “METTL3-independent m^6^A peaks” [35,52,61]. However, several studies have shown that even in *METTL3* knockout cells where m^6^A cannot be detected, m^6^A peaks can still be observed [62–64]. Thus, these m^6^A peaks should be regarded as background noise, rather than “METTL3-independent m^6^A peaks.” This noise can be due to m^6^A antibodies binding to non-m^6^A sites [60,61,63] or due to nonspecific binding during immunoprecipitation [65]. Thus, peaks that remain after *METTL3* knockout likely do not reflect real m^6^A sites. To identify true METTL3-independent m^6^A, methods such as SCARLET [66] can be used to validate that an m^6^A site in *METTL3* knockout actually reflects m^6^A.

A very small amount of m^6^A clearly remains after *Mettl3* knockout in both the exon4 *Mettl3* KO mESCs and in our conditional *Mettl3* KO MEFs. This m^6^A appears to be resistant to the METTL3 inhibitor STM2457. An alternative methyltransferase may be able to produce m^6^A on a small number of mRNA transcripts. For example, METTL16, which catalyzes the formation of m^6^A in U6 snRNA, also methylates *MAT2A* mRNA [67,68]. However, the sequence context of METTL16 is CAG [69–71], which differs from the predominant DRACH (D = A/G/U, R = A/G, H = A/C/U) sequence context for METTL3 [60,72,73]. Furthermore, METTL16 methylation is dependent on a hairpin structure found in U6 snRNA and *MAT2A*. Although current attempts to find other METTL16-dependent m^6^A in mRNA have been unsuccessful [61,68], METTL16-dependent m^6^A can likely be recognized based on the CAG sequence context and the U6-like hairpin structural context. m^6^A can also be formed by METTL5-TRMT112 and ZCCHC4 in the 18S and 28S rRNA, respectively [74,75]. Although current attempts have failed to identify m^6^A in mRNA catalyzed by these enzymes [74], a candidate m^6^A mediated by either of these enzymes will likely be in an AAC sequence context as well as the unique structural context utilized by these enzymes [74,76]. Any m^6^A predicted to be “METTL3-independent” is likely to exist within these unique structural contexts and would be lost upon depletion of one of these enzymes.

Importantly, our data does not negate the conclusions of the original studies by Batista and colleagues on the role of METTL3 in embryonic stem cell differentiation. Batista and colleagues used their *Mettl3* hypomorphic mESCs to show that m^6^A is required for differentiation from the naïve pluripotent stem cell state [4]. This result was later corroborated by Geula and colleagues using complete *Mettl3* knockout mESCs [5]. Although one should approach the initial study with caution due to the unknown effects of the hypomorphs, the study supports the idea that a partial depletion of m^6^A is sufficient to block differentiation of primed mESCs [5]. Thus, mESCs are highly sensitive to m^6^A levels and fail to differentiate even with partial loss of m^6^A. This has important implications for researchers attempting to inhibit METTL3 to influence cellular differentiation states, for example, in cancer [7,40].

## Materials and methods

### Cell culture

Exon2 *Mettl3* KO and WT mESCs were previously described by Batista and colleagues [4], and were a gift from P.J. Batista and H. Chang (Stanford University). Exon4 *Mettl3* KO and WT mESCs were previously described by Geula and colleagues [5], and were a gift from S. Geula and J.H. Hanna (Weizmann Institute of Science). All mESCs were grown in gelatinized (0.1% gelatin in water, EmbryoMax ES-006-B) tissue culture plates in mESC media (KnockOut DMEM (Gibco #10829018), 15% heat-inactivated fetal bovine serum (FBS) (Gibco #26140079), 100 U/ml penicillin, 100 μg/ml streptomycin (Gibco #15140122), 1× GlutaMax (Gibco #35050061), 55 μM β-mercaptoethanol (Gibco #21985023), 1× MEM non-essential amino acids (Gibco #11140076), 1,000 U/ml LIF (Millipore ESG1107), 3 μM CHIR99201 (Sigma Aldrich SML1046), 1 μM PD0325901 (APExBIO A3013)).

*METTL3* KO U2OS cells were previously described by Xiang and colleagues [23], and were a gift from Y. Xiang and Y. Shi (University of Oxford). *METTL3* KO A549 cells were previously described by Courtney and colleagues [41], and were a gift from D.G. Courtney and B.R. Cullen (Duke University Medical Center). U2OS cells and A549 cells were grown in high glucose DMEM (Gibco #11995073) with 10% FBS (Gibco #26140079) and 1% penicillin-streptomycin (Gibco #15140122).

All cells were grown at 37°C, 5.0% CO_2_. All cells were passaged as needed using TrypLE Express (Gibco #12604013).

### Mass spectrometry measurements of m^6^A

m^6^A measurements were performed using mass spectrometry as previously described [37]. Total RNA was extracted from cells using TRIzol Reagent (Invitrogen #15596026) and treated with 2 U RNase-free DNase I (New England Biolabs M0303) for 1 h. RNA was cleaned up using RNA Clean & Concentrator (Zymo R1017). RNA was decapped using yDcpS enzyme (New England Biolabs M0463) to release m^7^G caps as 7-methylguanosine-monophosphate (m^7^GMP), then digested using RNase T1 (Invitrogen AM2283) and S1 nuclease (Invitrogen #18001–016) in 1× mung bean nuclease buffer (New England Biolabs B0250) to release single nucleotides. Enzymes were precipitated out of solution using 4× volume of 100% methanol. No-RNAse T1 samples were also prepared and measured as background controls for m^6^A.

Nucleic acids were quantified by liquid chromatography with tandem mass spectrometry (LC–MS/MS) using a platform comprised of an Agilent Model 1290 Infinity II liquid chromatography system coupled to an Agilent 6460 Triple Quadrupole mass spectrometer equipped with Agilent Jet Stream Technology. Two injections of 2 μl each were performed for each sample, and the mean peak area was calculated from the 2 injections. m^6^A and m^7^GMP readings were taken from each injection. The m^6^A reading represents the amount of m^6^A in mRNA, while m^7^GMP readings represent the number of mRNAs in each sample. Mean m^6^A/m^7^GMP data of 2 or 3 biological replicates is reported with standard error. We thank N. Attarwala, Q.Y. Chen, and S.S. Gross (Weill Cornell Medicine) for conducting all LC–MS/MS experiments.

### Western blot

Cells were lysed in RIPA buffer (50 mM Tris-HCl (pH 7.5), 200 mM NaCl, 1% NP-40, 0.5% sodium deoxycholate, 0.1% SDS) with 1× Halt protease and phosphatase inhibitor cocktail (Thermo Scientific #78440). Cell debris was cleared by centrifuging at 14,000 G for 10 min. Protein quantification was done by Quick Start Bradford Protein assay (BioRad #5000201) or Pierce BCA protein assay kit (Thermo Scientific #23225) according to manufacturer’s instructions. Protein samples were resuspended in 1× NuPAGE LDS Sample Buffer (Invitrogen NP0007) + 50 mM dithiothreitol. Protein samples were separated by electrophoresis using NuPage 4% to 12% Bis-Tris gels (Invitrogen NP0322, NP0335) alongside Precision Plus Protein All Blue Prestained Protein Standards (Bio-Rad #1610373). Proteins were then transferred to nitrocellulose membranes in 1× Tris-glycine transfer buffer (25 mM Tris base, 200 mM glycine) + 20% methanol. Membranes were blocked using 5% BSA in phosphate buffered saline (PBS) + 0.1% (v/v) Tween-20 for 1 h at room temperature, then stained with primary antibodies overnight. Membranes were then washed with PBS + 0.1% (v/v) Tween-20, then stained with HRP-conjugated secondary antibodies for 1 h at room temperature. Membranes were washed with PBS + 0.1% (v/v) Tween-20 to remove excess antibody, and reactive bands were visualized using Pierce ECL Western Blotting Substrate (Thermo Scientific #32109) or SuperSignal West Femto Maximum Sensitivity Substrate (Thermo Scientific #34096). Western blots images were collected on a BioRad ChemiDoc XRS+ using the Image Lab software (BioRad).

### Antibodies

For immunoblotting experiments, we used anti-METTL3 rabbit polyclonal antibody (ProteinTech #15073-1-AP) directed against amino acids 229–580 in METTL3, anti-METTL3 rabbit monoclonal antibody (Cell Signalling Technology #96391S) directed against residues surrounding Leu297 of METTL3, and anti-GAPDH rabbit monoclonal antibody (Abcam ab181603) as primary antibodies. All primary antibodies were diluted 1:1,000 in 5% bovine serum albumin in PBS + 0.1% (v/v) Tween-20. Amersham ECL Rabbit IgG, HRP-linked whole antibody from donkey (Cytiva NA934) was used as secondary antibody, at 1:5,000 dilution in 5% bovine serum albumin in PBS + 0.1% (v/v) Tween-20.

### 5′ RACE

5′ RACE was performed using the Template Switching RT Enzyme Mix (New England Biolabs M0466) according to manufacturer’s instruction, and 1 μg total RNA was incubated with 10 mM dNTP and 10 mM dT(40) primer at 75°C for 5 min to allow the primer to hybridize. Full-length cDNA was reverse transcribed in 1× template switching RT buffer, 1× template switching RT enzyme, with 3.75 μM template switching oligo (GCT AAT CAT TGC AAG GAT CCG TAT CAA CGC AGA GTA CAT rGrGrG) for 90 min at 42°C. RNA was degraded using 5 units RNase H (New England Biolabs M0297) for 30 min. Approximately 10% of the cDNA product was amplified via PCR using a forward primer against the template switching oligo (CAT TGC AAG GAT CCG TAT CAA C, underlined = BamHI cut site) and a reverse primer against *Mettl3* exon 6 (CCA GGT AGC GGA TAT CAC AAC, underlined = EcoRV cut site). PCR amplification was performed in 20 μl 1× Phusion High-Fidelity PCR Master Mix with HF Buffer (NEB M0531) and using touchdown PCR cycling at 98°C for 30 s; 5 cycles of 98°C for 10 s, 72°C for 60 s; 5 cycles of 98°C for 10 s, 70°C for 60 s; 27 cycles of 98°C for 10 s, 65°C for 30 s, 72°C for 60 s; 72°C for 10 min; 4°C hold.

The 5′ RACE PCR product was digested using BamHI (New England Biolabs R0136) and EcoRV (New England Biolabs R3195) cut sites found in the primers and cloned into a pcDNA3.1(+) backbone using the Quick Ligation Kit (New England Biolabs M2200). Plasmids were transformed and grown in subcloning efficiency DH5α competent cells (Invitrogen #18265017) before extraction by Miniprep (QIAGEN #27104) and Sanger sequencing. Sequences of the 5′ RACE products can be found in S1 Table.

### Cloning and transfection of METTL3 isoform ORFs

ORFs of METTL3 isoforms were identified from the *Mettl3* mRNAs by finding the longest ORF that encoded an AUG start codon, a protein which aligned with the METTL3 protein, and which was not interrupted by premature stop codons. The full sequence of the METTL3 isoform ORFs identified can be found in S2 Table.

The ORFs for full-length METTL3, METTL3-a.i, and METTL3-b.i were constructed via PCR from WT *Mettl3* cDNA. FLAG-tags were included at the N-terminus. Primers used for PCR of the METTL3 isoform ORFs can be found in S5 Table. PCR amplification was performed in 1× Phusion High-Fidelity PCR Master Mix with HF Buffer (New England Biolabs M0531) and PCR cycling at 98°C for 30 s; 25 cycles of 98°C for 10 s, 65°C for 30 s, 72°C for 90 s; 72°C for 10 min; 4°C hold. The ORFs were then digested using HindIII (New England Biolabs R0104) and BamHI (New England Biolabs R0136), and cloned into a pcDNA3.1(+) vector using the Quick Ligation Kit (New England Biolabs M2200). All constructs were verified to be identical to the *Mettl3* mRNAs from exon2 *Mettl3* KO mESCs via Sanger sequencing.

Exon-skipping ORFs METTL3-a.ii and METTL3-b.ii were constructed via PCR from WT *Mettl3* cDNA, followed by assembly and cloning into a pcDNA3.1(+) backbone using Gibson Assembly (New England Biolabs E2611). FLAG-tags were included at the N-terminus. Primers used for PCR of the METTL3 isoform ORFs can be found in S5 Table. PCR amplification was performed in 1× Phusion High-Fidelity PCR Master Mix with HF Buffer (NEB M0531) and PCR cycling at 98°C for 30 s; 25 cycles of 98°C for 10 s, 65°C for 30 s, 72°C for 90 s; 72°C for 10 min; 4°C hold. Gibson assembly was performed in 1× Gibson Assembly Master Mix (New England Biolabs E2611) for 1 h at 50°C with 1:5 ratio of vector to inserts. All constructs were verified to be identical to the *Mettl3* mRNAs from exon2 *Mettl3* KO mESCs via Sanger sequencing.

Plasmids were transformed and grown in high efficiency DH5α competent cells (New England Biolabs C2987) before extraction by Miniprep (QIAGEN #27104). Sequences were confirmed via Sanger sequencing.

Plasmids were transfected into mESCs using FuGENE HD transfection reagent (Promega E2311). Exon4 WT mESCs or *Mettl3* KO mESCs were plated at 150,000 cells/well on gelatinized (0.1% gelatin in water, EmbryoMax ES-006-B) 6-well plates. The next day, when the mESCs were at 50% confluency, cells were transfected with 2.5 μg of METTL3 isoform ORF-expressing plasmids using 7.5 μl FuGene HD reagent per well. After 48 h of transfection, cell lysate was collected for western blot, and RNA was extracted for m^6^A measurements. Successful transfection was confirmed via western blot using anti-FLAG antibody and anti-METTL3 antibody.

### Polysome profiling of *Mettl3* KO cells

Polysomes were isolated from exon2 *Mettl3* KO mESCs as follows: cells were lysed in lysis buffer (20 mM Tris-HCL (pH 7.4), 100 mM KCl, 5 mM MgCl_2,_ 1% Triton-X 100, 100 μg/ml cycloheximide, 2 mM DTT, 1× Halt Protease Inhibitor (Thermo Scientific #78430)) by passing through a 25 G 1 ½ syringe 10 times. A total of 130 μg of RNA was used per sample. Polysomes were separated using a 10% to 50% sucrose gradient and collected using the Piston Gradient Fractionator (BioComp). Fractions were pooled together as described in S3 Fig, and RNA was extracted using TRIzol LS (Invitrogen #10296010) with 10 mM EDTA to disassemble polysomes.

To identify *Mettl3* mRNAs in the polysome fractions, RNA from each fraction was reverse transcribed using SuperScriptIV (Invitrogen #18090010) and a primer against the most 3′ end of the *Mettl3* transcript (S5 Table). PCR amplification was performed using primers against the most 5′ end and the most 3′ end of the *Mettl3* transcript (S5 Table) in 1× Phusion High-Fidelity PCR Master Mix with HF Buffer (New England Biolabs M0531) and PCR cycling at 98°C for 30 s; 30 cycles of 98°C for 10 s, 65°C for 30 s, 72°C for 90 s; 72°C for 10 min; 4°C hold. PCR products were digested using BamHI (New England Biolabs R0136) and HindIII (New England Biolabs R0104) cut sites found in the primers and cloned into a pcDNA3.1(+) backbone using the Quick Ligation Kit (New England Biolabs M2200). Plasmids were transformed and grown in subcloning efficiency DH5α competent cells (Invitrogen #18265017) before extraction by Miniprep (QIAGEN #27104) and Sanger sequencing.

### STM2457 inhibition of METTL3

Cells were seeded to be 50% confluent. The next day, cells were treated with 30 μM STM2457 or with equal volume DMSO (negative control). After 48 h of treatment, total RNA was extracted from cells using TRIzol Reagent (Invitrogen #15596026) for m^6^A measurements.

### 2D-TLC measurement of m^6^A

m^6^A levels in mRNA were measured by 2D-TLC (2-dimensional thin-layer chromatography) as described previously [77]. m^6^A was poly-A selected twice using Dynabeads Oligo(dT)_25_ (Invitrogen #61002) according to manufacturer’s protocol to remove potential contamination from ribosomal RNAs or other noncoding RNAs. A total of 100 ng of twice poly-A-selected RNA was then digested by 1 U RNase T1 (Invitrogen AM2283) in 1× PNK buffer for 2 h at 37°C. This cuts mRNA after G, therefore only exposing m^6^As in a GA context and omitting m^6^As in the poly-A tail or in other non-mRNA contexts. The 5′ end of the digested RNA is then labeled with 10 U T4 PNK (New England Biolabs M0201) and 10 μCi [γ-^32^P]ATP (Perkin Elmer BLU002Z250UC) for 30 min at 37°C; γ-phosphate was then removed from excess [γ-^32^P]ATP with 0.5 U apyrase (New England Biolabs M0398) in 1× apyrase buffer for 10 min at 30°C. RNA was then digested to single nucleotides using 2 U Nuclease P1 (FUJIFILM Wako Pure Chemical Corporation #145–08221) for 1 h at 60°C.

2 μl of the digested RNA is spotted on PEI-cellulose TLC plates (Millipore Sigma #105579) 0.5 μl at a time. Plates were developed in 5:3 (v/v) isobutyric acid:0.5 M NH_4_OH in the first dimension, then in 70:15:15 (v/v/v) isopropanol:water:HCl in the second dimension. Radioactively labeled nucleotides were detected using a phosphor storage screen and Amersham Biosciences Typhoon 9400 Variable Mode Imager. m^6^A levels were quantified using Image Lab software (BioRad).

### DepMap dataset analysis

Gene dependency probability scores were obtained from the DepMap Public 21Q4 CRISPR gene dependency dataset [43–47]. We extracted the gene dependency probability scores of 1,054 cell lines on the gene *METTL3* and plotted them to identify cell lines that were dependent on *METTL3*. Gene dependency probability scores greater than 0.5 indicate that a cell line is dependent on the gene. To account for off-target effects, we extracted the gene dependency scores of 1,054 cell lines on *METTL3*, *METTL14*, and *WTAP*. We identified cell lines that were dependent on all of these genes (gene dependency probability scores >0.5).

To identify cell lines that may be independent of m^6^A, we extracted the gene dependency scores of 1,054 cell lines on *METTL3*, *METTL14*, and *WTAP*. We identified cell lines that were independent of each of these genes (gene dependency probability scores <0.5). We next identified cell lines that were independent of all 3 genes to obtain a subset of cell lines that may be independent of m^6^A. The full list of predicted m^6^A-independent cell lines can be found in S4 Table.

### Generation of *Mettl3* conditional KO MEFs

*Mettl3*^*flox/flox*^ mice were generated based on construction from the Knockout Mouse Project Repository (KOMP) and were described by Cheng and colleagues [32]. *loxP* sites were inserted spanning the fourth exon of *Mettl3*.

To generate tamoxifen-inducible *Mettl3* KO MEFs, embryos from *Mettl3*^*flox/flox*^ mice were collected on day 13.5, mechanically separated, trypsinized, and plated. After 3 passages, cells were transduced with SV40 large T-antigen and passaged until they reached senescence. Cells that escaped senescence and became immortalized were then infected with Cre-ERT2 lentivirus. Successfully infected cells were selected with puromycin. Single-cell colonies were then isolated and expanded.

To induce the *Mettl3* knockout, MEFs were plated at 100,000 cells/well in a 12-well plate. The next day, when the MEFs were at 50% confluency, cells were treated with 4-hydroxytamoxifen (500 nM) or ethanol (negative control) for 48 h. Cells continued to be grown and passaged normally after 4-hydroxytamoxifen treatment. Cell lysate was collected 5 days after tamoxifen treatment for western blot. RNA was collected 8 days after tamoxifen treatment for m^6^A measurements.

### MTT assay

To measure the changes in cell proliferation upon *Mettl3* KO, tamoxifen-inducible *Mettl3* KO MEFs were plated at 5,000 cells/well in a 12-well plate. The next day, cells were treated with 4-hydroxytamoxifen (500 nM) or ethanol (negative control). Treatment continued for the duration of the experiment, and then, 0, 2, 4, 6, and 8 days after 4-hydroxytamoxifen treatment, cells were washed with PBS and then incubated in 1:1 (v/v) high-glucose DMEM without phenol red (Gibco #21063029) and MTT (3-(4,5-dimethylthiazol-2-yl)-2,5-diphenyltetrazolium bromide) reagent (5 mg/ml MTT (Abcam ab146345) in PBS) for 3 h at 37°C. The produced formazan crystals were dissolved in 1.5× volume MTT solvent (4 mM HCl, 0.1% NP-40 in isopropanol) and incubated for 15 min at room temperature while shaking on an orbital shaker. Samples without cells were included as background controls. Absorbance was read at 570 nM to estimate the number of cells.

## Supporting information

S1 Fig Exon2 *Mettl3* KO mESCs express altered METTL3 proteins while exon4 *Mettl3* KO mESCs do not. (**A**) Exon2 *Mettl3* KO mESCs express a novel protein that is immunoreactive to several anti-METTL3 antibodies. We found that exon2 *Mettl3* KO mESCs express new proteins that are reactive to an anti-METTL3 pAb. To confirm that these new proteins may be shortened versions of METTL3, we performed a second WB with a different anti-METTL3 mAb. Similar to the first WB, we found that full-length METTL3 (75 kDa, arrowhead) was lost in both KO cell lines. In the KO cell lines, the same bands that were immunoreactive to the polyclonal anti-METTL3 antibody were also immunoreactive to the anti-METTL3 mAb at approximately 50 kDa in *Mettl3* KO mESC-a and approximately 55 kDa in *Mettl3* KO mESC-b, respectively (arrowheads). This further validates the possibility that the *Mettl3* KO mESCs express smaller versions of METTL3 proteins. 30 μg per lane. (**B**) Exon4 *Mettl3* KO mESCs do not express METTL3 protein. An independently run replicate of the WB from Fig 1B reveals that exon4 *Mettl3* KO mESCs do not express any proteins immunoreactive to anti-METTL3 antibodies. 30 μg per lane. (**C**) Exon4 *Mettl3* KO mESCs do not express METTL3 protein. To confirm that exon4 *Mettl3* KO mESCs do not express METTL3, we performed a second WB with a different anti-METTL3 mAb. We confirmed that exon4 *Mettl3* KO mESCs indeed do not express any detectable METTL3 protein. 30 μg per lane. mAb, monoclonal antibody; mESC, mouse embryonic stem cell; pAb, polyclonal antibody; WB, western blot; WT, wild-type.
(TIFF)

S2 Fig Mouse embryonic stem cells are transfected at low efficiency. Transfection efficiency of mESCs is low. Optimization of transfection conditions using plasmids expressing eGFP showed that only 10% to 30% of mESCs become successfully transfected and express eGFP after 48 h. This suggests that only a small number of cells will express proteins after plasmid transfection, and may explain the low level of m^6^A rescue after transfection of exon4 *Mettl3* KO mESCs with WT METTL3 (Fig 2C). eGFP, enhanced green fluorescent protein; mESC, mouse embryonic stem cell; WT, wild-type.
(TIFF)

S3 Fig Polysome profiling reveals that exon2 *Mettl3* KO mESCs translate *Mettl3* mRNAs. To investigate if the altered *Mettl3* mRNAs expressed in the exon2 *Mettl3* KO mESCs are translated, we performed polysome profiling. The polysomes were separated into 4 fractions based on their ribosome load: Fraction 1 (sub-monosomal), Fraction 2 (low polysomes), Fraction 3 (medium polysomes), and Fraction 4 (high polysomes). RT-PCR of *Mettl3* using primers against the 5′ and 3′ end of *Mettl3* revealed *Mettl3* mRNAs (arrowheads) in the medium and high polysome fractions in the WT mESCs, as well as the exon2 *Mettl3* KO mESCs. Sequencing of the PCR products revealed *Mettl3* mRNAs depicted in blue. They include *mRNA-3* that encodes METTL3-a.ii in exon2 *Mettl3* KO mESC-a, and *mRNA-7* that encodes METTL3-b.ii in exon 2 *Mettl3* KO mESC-b (S3 Table). This suggests that the *Mettl3* mRNAs being expressed can indeed be translated to produce the METTL3 proteins we see in these *Mettl3* KO cell lines. Underlying data can be found in S1 Data. mESC, mouse embryonic stem cell; RT-PCR, reverse transcription PCR; WT, wild-type.
(TIFF)

S4 Fig STM2457 treatment has no effect on m^6^A in exon4 *Mettl3* KO mESCs. Treatment of exon4 *Mettl3* KO mESCs with the METTL3-specific inhibitor STM2457 does not reduce m^6^A levels. Treatment of the parental WT mESC cell line with 30 μM of STM2457 lead to 65.4% reduction in m^6^A levels. On the other hand, STM2457 treatment did not lead to a significant change in m^6^A levels in exon4 *Mettl3* KO mESCs, indicating that the small amount of remaining m^6^A was not produced by METTL3. Error bars indicate standard error (*n* = 3). * = *p*-value < 0.5, ** = *p*-value < 0.01, *** = *p*-value < 0.005, n.s. = not significant. Underlying data can be found in S1 Data. m^6^A, *N*^6^-methyladenosine; m^7^GMP, 7-methylguanosine monophosphate; mESC, mouse embryonic stem cell; WT, wild-type.
(TIFF)

S5 Fig *METTL3* KO A549 cells have high m^6^A levels. (**A**) *METTL3* KO A549 cells have persistent m^6^A. *METTL3* KO A549 cells were previously reported [38]. To find out how much m^6^A remains in these cells, we measured the levels of m^6^A using 2D-TLC that measures m^6^A specifically in the GA context [77]. This limits the detection of m^6^A to only m^6^A in mRNAs, where they are found in a DRACH context [58,71,72]. The m^6^A level in the *METTL3* KO A549 cells was very similar to m^6^A levels in WT A549 cells. This suggests that either the knockout of *METTL3* was incomplete or that A549 cells may express a non-METTL3 m^6^A methyltransferase that is responsible for the majority of m^6^A in mRNAs (*n* = 1). Underlying data can be found in S1 Data. 2D-TLC, 2-dimensional thin-layer chromatography; m^6^A, *N*^6^-methyladenosine; WT, wild-type.
(TIFF)

S6 Fig A large number of cell lines are dependent on the m^6^A methyltransferase complex components *METTL3*, *METTL14*, and *WTAP*. A large number of cell lines are dependent on the components of the m^6^A methyltransferase complex for proliferation. Using the DepMap CRISPR loss-of-function screening dataset, we found that 801 of 1,054 tested cell lines require *METTL3* for proliferation (Fig 4A). In order to assess the influence of off-target effects on this result, we further analyzed the dataset to find cell lines that are dependent on other members of the m^6^A methyltransferase complex, *WTAP*, and *METTL14*. We find that 683 of the 801 *METTL3*-dependent cell lines are also dependent on both *METTL14* and *WTAP*. The large overlap in cell lines dependent on the m^6^A methyltransferase complex members suggests that these cell lines are truly dependent on m^6^A methyltransferase activity, and the contribution of off-target effects in the CRISPR screen for *METTL3* dependence is low. m^6^A, *N*^6^-methyladenosine.
(TIFF)

S7 Fig Depletion of METTL3 leads to slower proliferation in MEFs. (**A**) *Mettl3* conditional knockout MEFs do not express METTL3 protein. To confirm that *Mettl3* is the sole m^6^A writer in cell lines other than mESCs, we produced a tamoxifen-inducible *Mettl3* conditional knockout MEF cell line. We used a WB to confirm the loss of METTL3 using a second anti-METTL3 mAb. Five days after 4OHT treatment (500 nM), we observe loss of the WT METTL3 protein. 30 μg per lane. (**B**) *Mettl3* knockout in MEFs leads to a decrease in cellular proliferation. Using an MTT assay, we measured cell proliferation after 4OHT-induced *METTL3* knockout over 8 days. Proliferation of *Mettl3* KO MEFs began to slow down compared to WT MEFs after 6 days of 4OHT treatment. Error bars indicate standard error (*n* = 3). * = *p*-value < 0.5, ** = *p*-value < 0.01, *** = *p*-value < 0.005, n.s. = not significant. Underlying data can be found in S1 Data. mAb, monoclonal antibody; m^6^A, *N*^6^-methyladenosine; MEF, mouse embryonic fibroblast; mESC, mouse embryonic stem cell; WB, western blot; 4OHT, 4-hydroxytamoxifen.
(TIFF)

S1 Table Sequences of 5′ RACE products from *Mettl3* in exon2 *Mettl3* KO mESCs. mESC, mouse embryonic stem cell; RACE, rapid amplification of cDNA ends. (XLSX)

S2 Table Sequences of METTL3 ORFs identified in exon2 *Mettl3* KO mESCs. mESC, mouse embryonic stem cell; ORF, open reading frame. (XLSX)

S3 Table Sequences of *Mettl3* mRNAs identified in polysome fractions. (XLSX)

S4 Table List of cell lines predicted to be m^6^A-independent. m^6^A, *N*^6^-methyladenosine. (CSV)

S5 Table Primers used in this manuscript. (XLSX)

S1 Data Underlying data for figures in this manuscript. (XLSX)

S1 Raw images Raw images for all gels and Western blots in this manuscript. (PDF)

## Abbreviations

DepMap: Dependency Map Project
FBS: fetal bovine serum
m^6^A: *N*^6^-methyladenosine
MEF: mouse embryonic fibroblast
mESC: mouse embryonic stem cell
ORF: open reading frame
PBS: phosphate buffered saline
RACE: rapid amplification of cDNA ends
WT: wild-type
ZFD: zinc finger domain
