# Supplementary figures and images for: Alternative splicing of METTL3 explains apparently METTL3-independent m6A modifications in mRNA
Source: PLoS Biol. 2022 Jul 19;20(7):e3001683. doi: 10.1371/journal.pbio.3001683 (PMC9295969; doi:10.1371/journal.pbio.3001683)

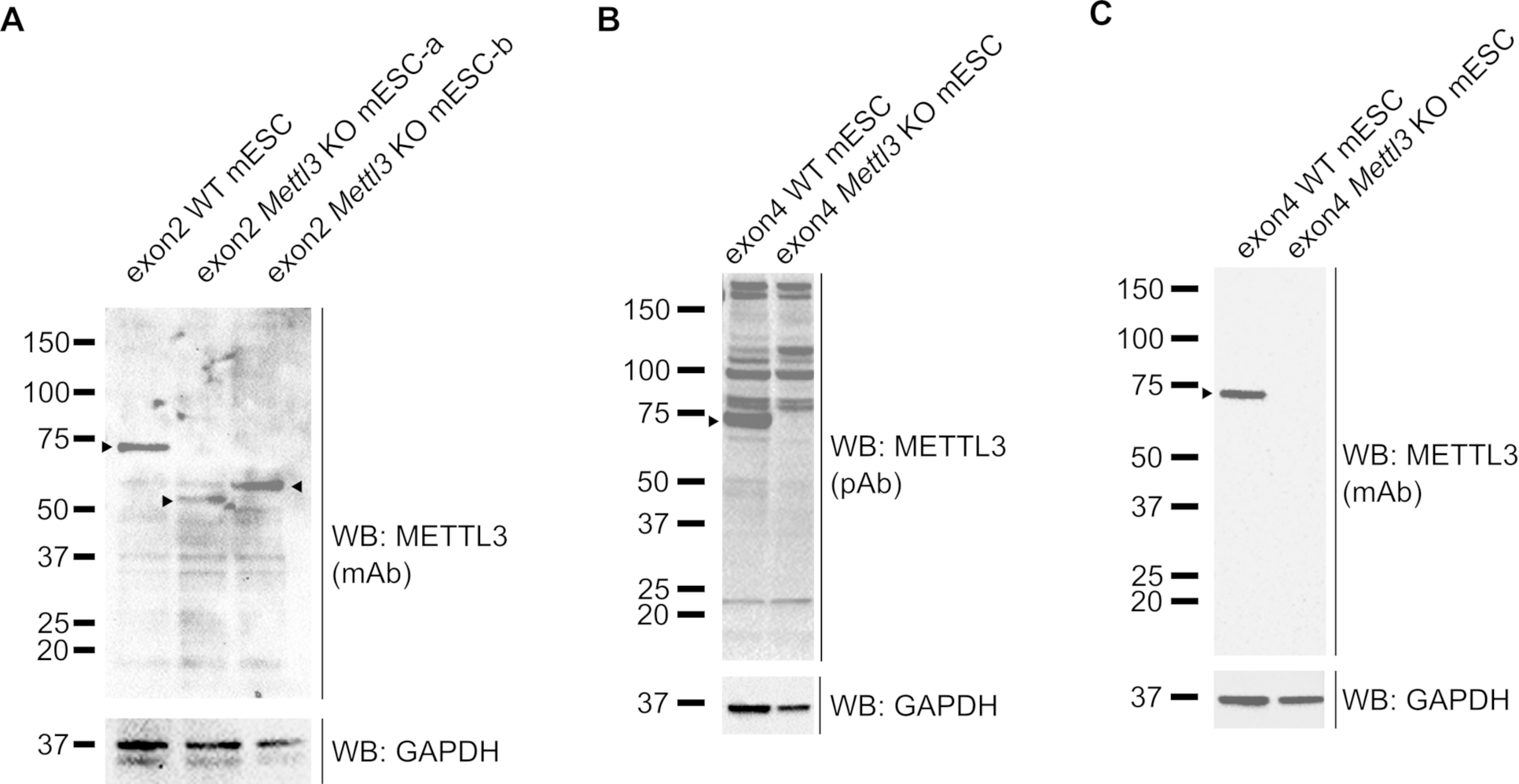

Supplement: S1 Fig — (A) Exon2 Mettl3 KO mESCs express a novel protein that is immunoreactive to several anti-METTL3 antibodies. We found that exon2 Mettl3 KO mESCs express new proteins that are reactive to an anti-METTL3 pAb. To confirm that these new proteins may be shortened versions of METTL3, we performed a second WB with a different anti-METTL3 mAb. Similar to the first WB, we found that full-length METTL3 (75 kDa, arrowhead) was lost in both KO cell lines. In the KO cell lines, the same bands that were immunoreactive to the polyclonal anti-METTL3 antibody were also immunoreactive to the anti-METTL3 mAb at approximately 50 kDa in Mettl3 KO mESC-a and approximately 55 kDa in Mettl3 KO mESC-b, respectively (arrowheads). This further validates the possibility that the Mettl3 KO mESCs express smaller versions of METTL3 proteins. 30 μg per lane. (B) Exon4 Mettl3 KO mESCs do not express METTL3 protein. An independently run replicate of the WB from Fig 1B reveals that exon4 Mettl3 KO mESCs do not express any proteins immunoreactive to anti-METTL3 antibodies. 30 μg per lane. (C) Exon4 Mettl3 KO mESCs do not express METTL3 protein. To confirm that exon4 Mettl3 KO mESCs do not express METTL3, we performed a second WB with a different anti-METTL3 mAb. We confirmed that exon4 Mettl3 KO mESCs indeed do not express any detectable METTL3 protein. 30 μg per lane. mAb, monoclonal antibody; mESC, mouse embryonic stem cell; pAb, polyclonal antibody; WB, western blot; WT, wild-type. (TIFF) [file pbio.3001683.s001.tiff]

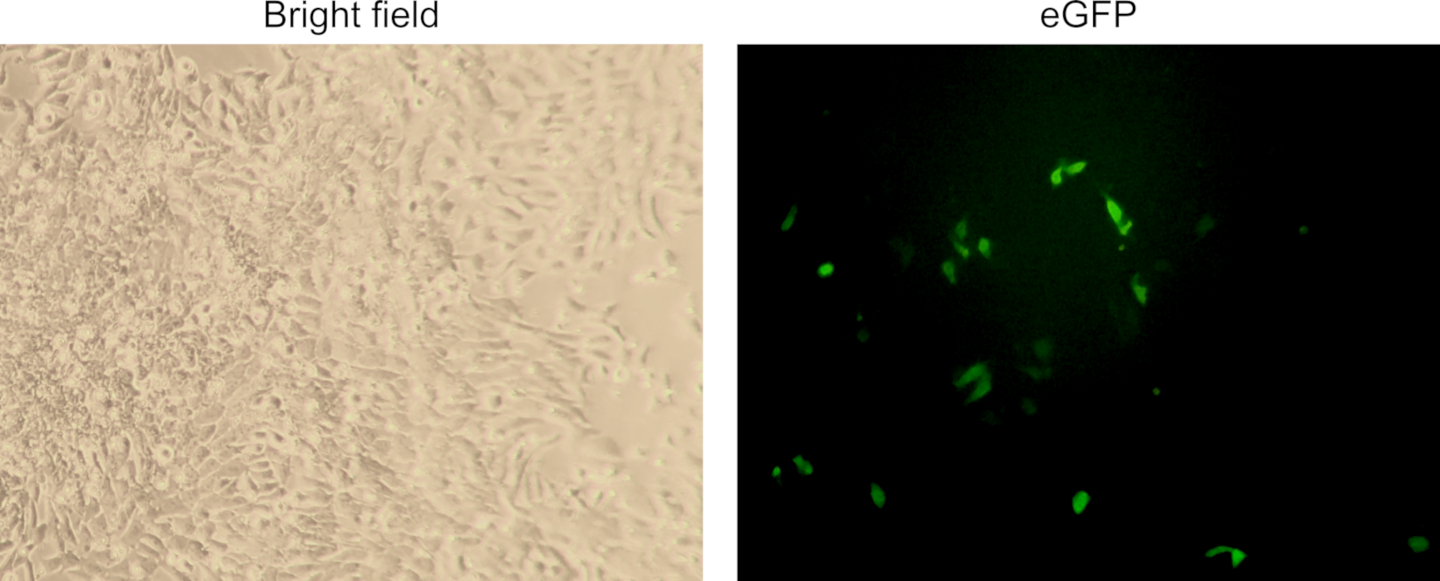

Supplement: S2 Fig — Transfection efficiency of mESCs is low. Optimization of transfection conditions using plasmids expressing eGFP showed that only 10% to 30% of mESCs become successfully transfected and express eGFP after 48 h. This suggests that only a small number of cells will express proteins after plasmid transfection, and may explain the low level of m6A rescue after transfection of exon4 Mettl3 KO mESCs with WT METTL3 (Fig 2C). eGFP, enhanced green fluorescent protein; mESC, mouse embryonic stem cell; WT, wild-type. (TIFF) [file pbio.3001683.s002.tiff]

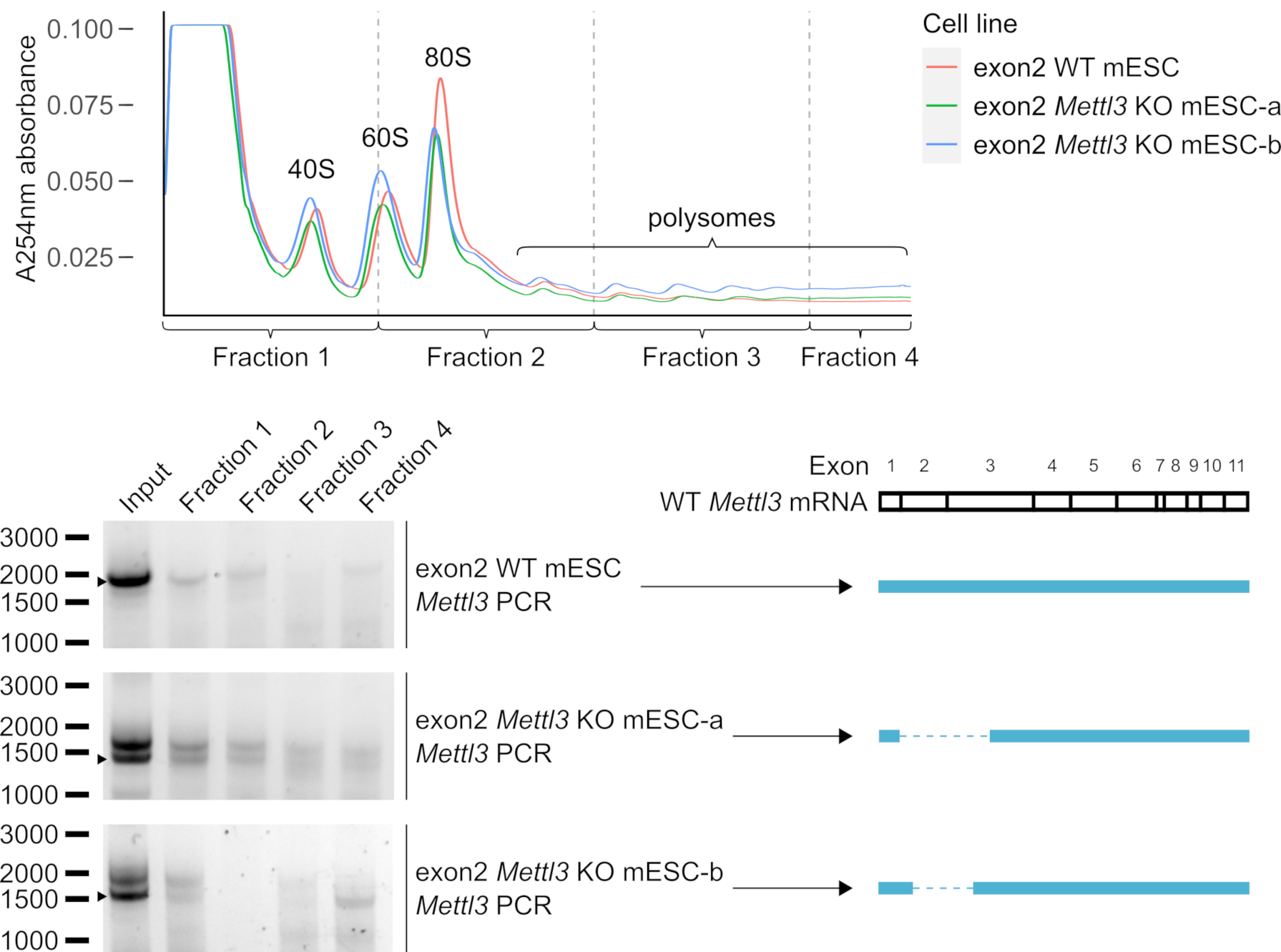

Supplement: S3 Fig — To investigate if the altered Mettl3 mRNAs expressed in the exon2 Mettl3 KO mESCs are translated, we performed polysome profiling. The polysomes were separated into 4 fractions based on their ribosome load: Fraction 1 (sub-monosomal), Fraction 2 (low polysomes), Fraction 3 (medium polysomes), and Fraction 4 (high polysomes). RT-PCR of Mettl3 using primers against the 5′ and 3′ end of Mettl3 revealed Mettl3 mRNAs (arrowheads) in the medium and high polysome fractions in the WT mESCs, as well as the exon2 Mettl3 KO mESCs. Sequencing of the PCR products revealed Mettl3 mRNAs depicted in blue. They include mRNA-3 that encodes METTL3-a.ii in exon2 Mettl3 KO mESC-a, and mRNA-7 that encodes METTL3-b.ii in exon 2 Mettl3 KO mESC-b (S3 Table). This suggests that the Mettl3 mRNAs being expressed can indeed be translated to produce the METTL3 proteins we see in these Mettl3 KO cell lines. Underlying data can be found in S1 Data. mESC, mouse embryonic stem cell; RT-PCR, reverse transcription PCR; WT, wild-type. (TIFF) [file pbio.3001683.s003.tiff]

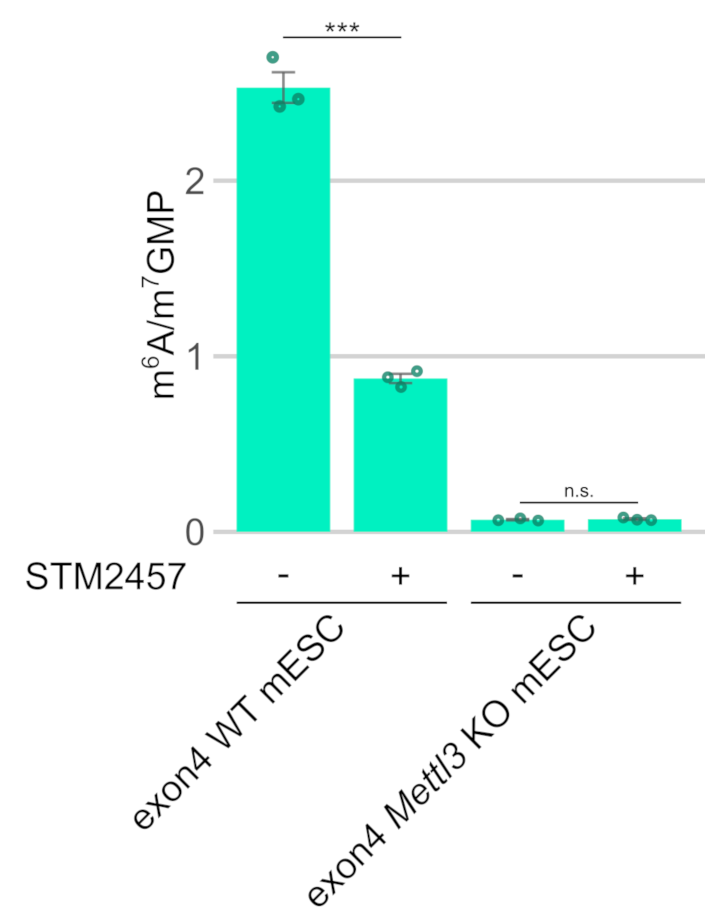

Supplement: S4 Fig — Treatment of exon4 Mettl3 KO mESCs with the METTL3-specific inhibitor STM2457 does not reduce m6A levels. Treatment of the parental WT mESC cell line with 30 μM of STM2457 lead to 65.4% reduction in m6A levels. On the other hand, STM2457 treatment did not lead to a significant change in m6A levels in exon4 Mettl3 KO mESCs, indicating that the small amount of remaining m6A was not produced by METTL3. Error bars indicate standard error (n = 3). * = p-value < 0.5, ** = p-value < 0.01, *** = p-value < 0.005, n.s. = not significant. Underlying data can be found in S1 Data. m6A, N6-methyladenosine; m7GMP, 7-methylguanosine monophosphate; mESC, mouse embryonic stem cell; WT, wild-type. (TIFF) [file pbio.3001683.s004.tiff]

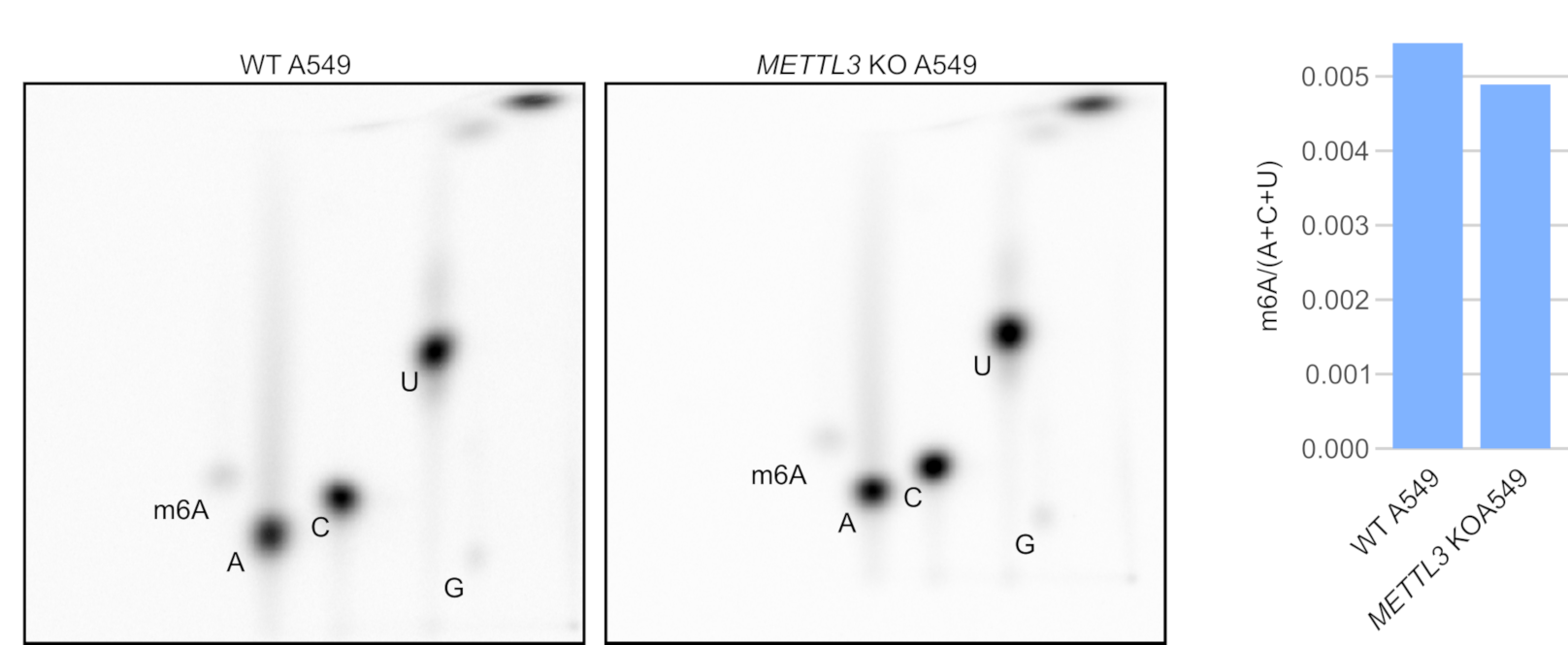

Supplement: S5 Fig — (A) METTL3 KO A549 cells have persistent m6A. METTL3 KO A549 cells were previously reported [38]. To find out how much m6A remains in these cells, we measured the levels of m6A using 2D-TLC that measures m6A specifically in the GA context [77]. This limits the detection of m6A to only m6A in mRNAs, where they are found in a DRACH context [58,71,72]. The m6A level in the METTL3 KO A549 cells was very similar to m6A levels in WT A549 cells. This suggests that either the knockout of METTL3 was incomplete or that A549 cells may express a non-METTL3 m6A methyltransferase that is responsible for the majority of m6A in mRNAs (n = 1). Underlying data can be found in S1 Data. 2D-TLC, 2-dimensional thin-layer chromatography; m6A, N6-methyladenosine; WT, wild-type. (TIFF) [file pbio.3001683.s005.tiff]

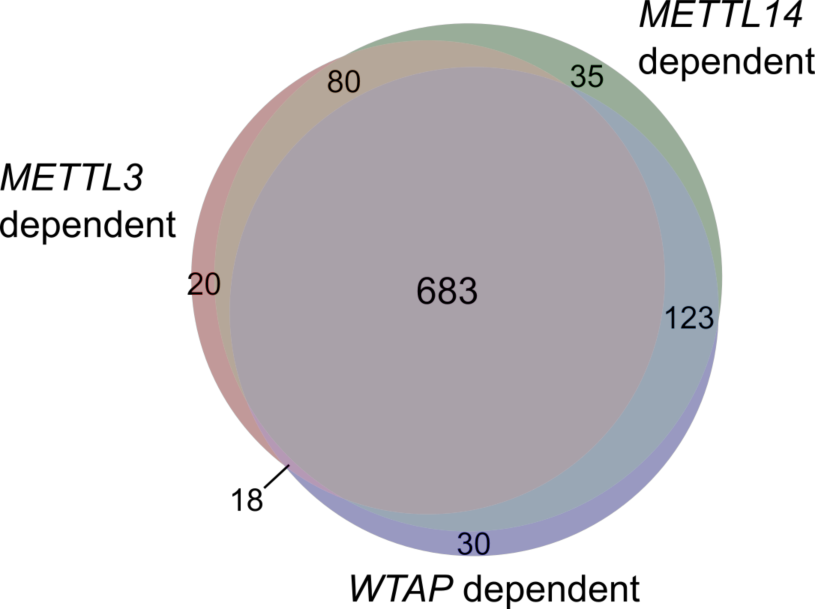

Supplement: S6 Fig — A large number of cell lines are dependent on the components of the m6A methyltransferase complex for proliferation. Using the DepMap CRISPR loss-of-function screening dataset, we found that 801 of 1,054 tested cell lines require METTL3 for proliferation (Fig 4A). In order to assess the influence of off-target effects on this result, we further analyzed the dataset to find cell lines that are dependent on other members of the m6A methyltransferase complex, WTAP, and METTL14. We find that 683 of the 801 METTL3-dependent cell lines are also dependent on both METTL14 and WTAP. The large overlap in cell lines dependent on the m6A methyltransferase complex members suggests that these cell lines are truly dependent on m6A methyltransferase activity, and the contribution of off-target effects in the CRISPR screen for METTL3 dependence is low. m6A, N6-methyladenosine. (TIFF) [file pbio.3001683.s006.tiff]

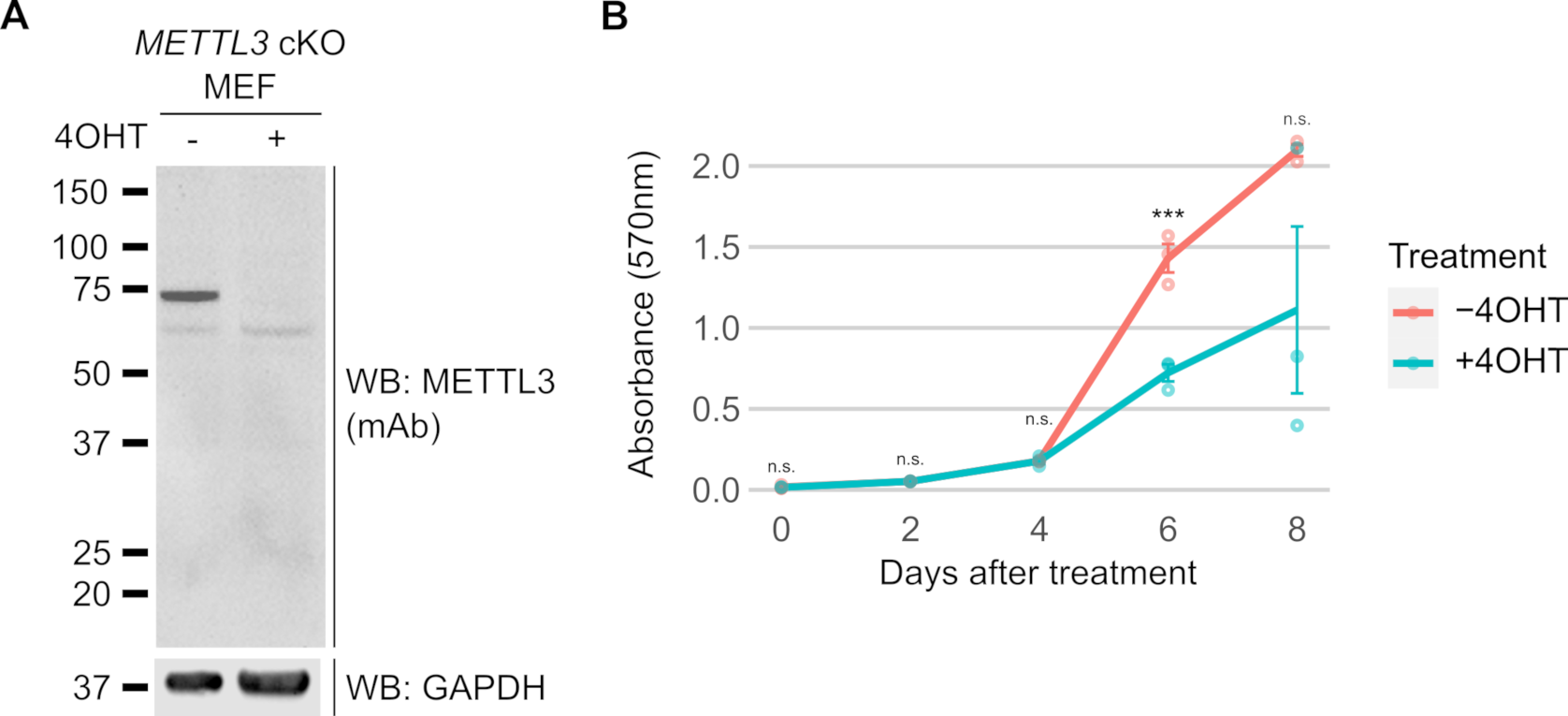

Supplement: S7 Fig — (A) Mettl3 conditional knockout MEFs do not express METTL3 protein. To confirm that Mettl3 is the sole m6A writer in cell lines other than mESCs, we produced a tamoxifen-inducible Mettl3 conditional knockout MEF cell line. We used a WB to confirm the loss of METTL3 using a second anti-METTL3 mAb. Five days after 4OHT treatment (500 nM), we observe loss of the WT METTL3 protein. 30 μg per lane. (B) Mettl3 knockout in MEFs leads to a decrease in cellular proliferation. Using an MTT assay, we measured cell proliferation after 4OHT-induced METTL3 knockout over 8 days. Proliferation of Mettl3 KO MEFs began to slow down compared to WT MEFs after 6 days of 4OHT treatment. Error bars indicate standard error (n = 3). * = p-value < 0.5, ** = p-value < 0.01, *** = p-value < 0.005, n.s. = not significant. Underlying data can be found in S1 Data. mAb, monoclonal antibody; m6A, N6-methyladenosine; MEF, mouse embryonic fibroblast; mESC, mouse embryonic stem cell; WB, western blot; 4OHT, 4-hydroxytamoxifen. (TIFF) [file pbio.3001683.s007.tiff]

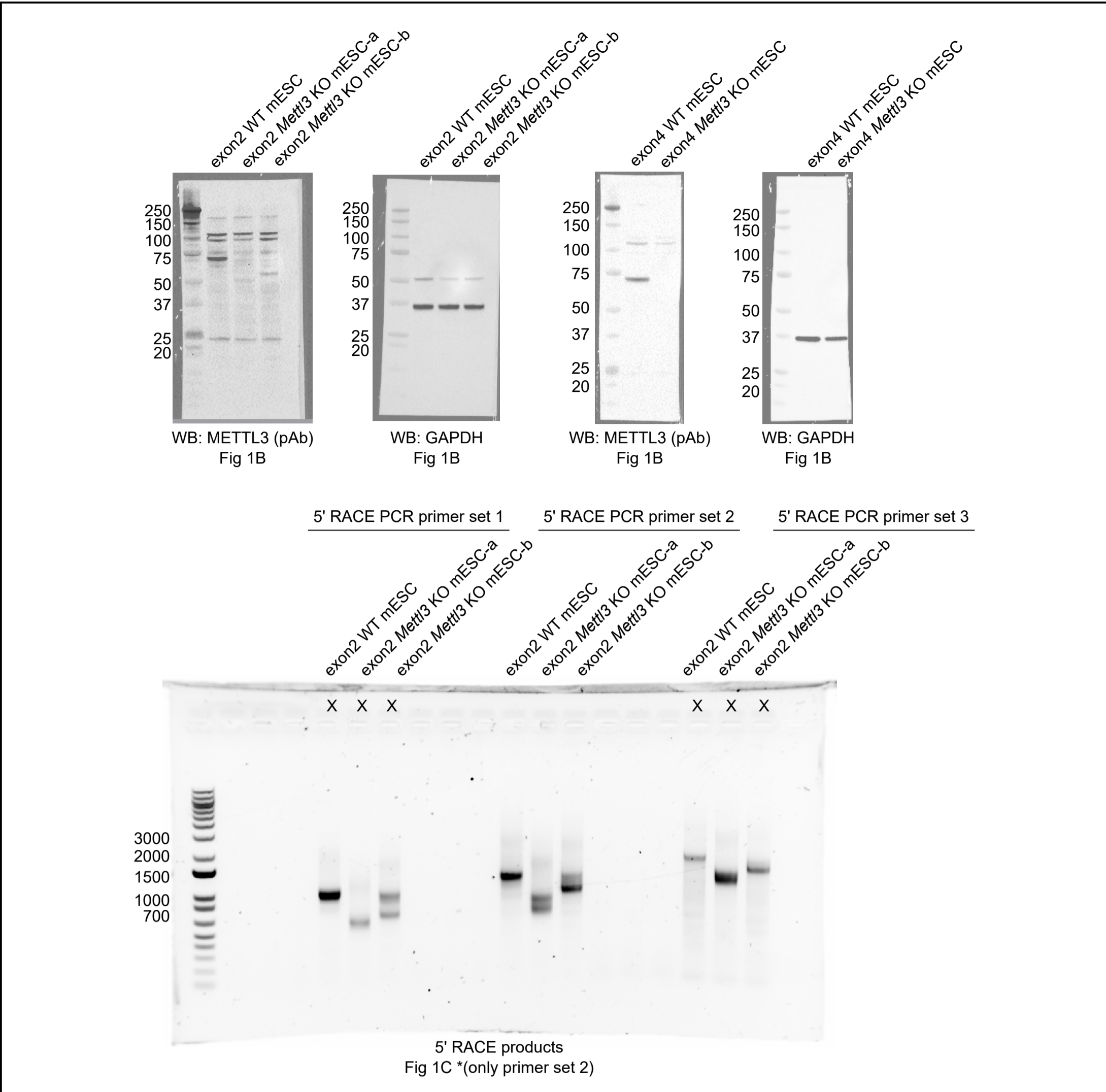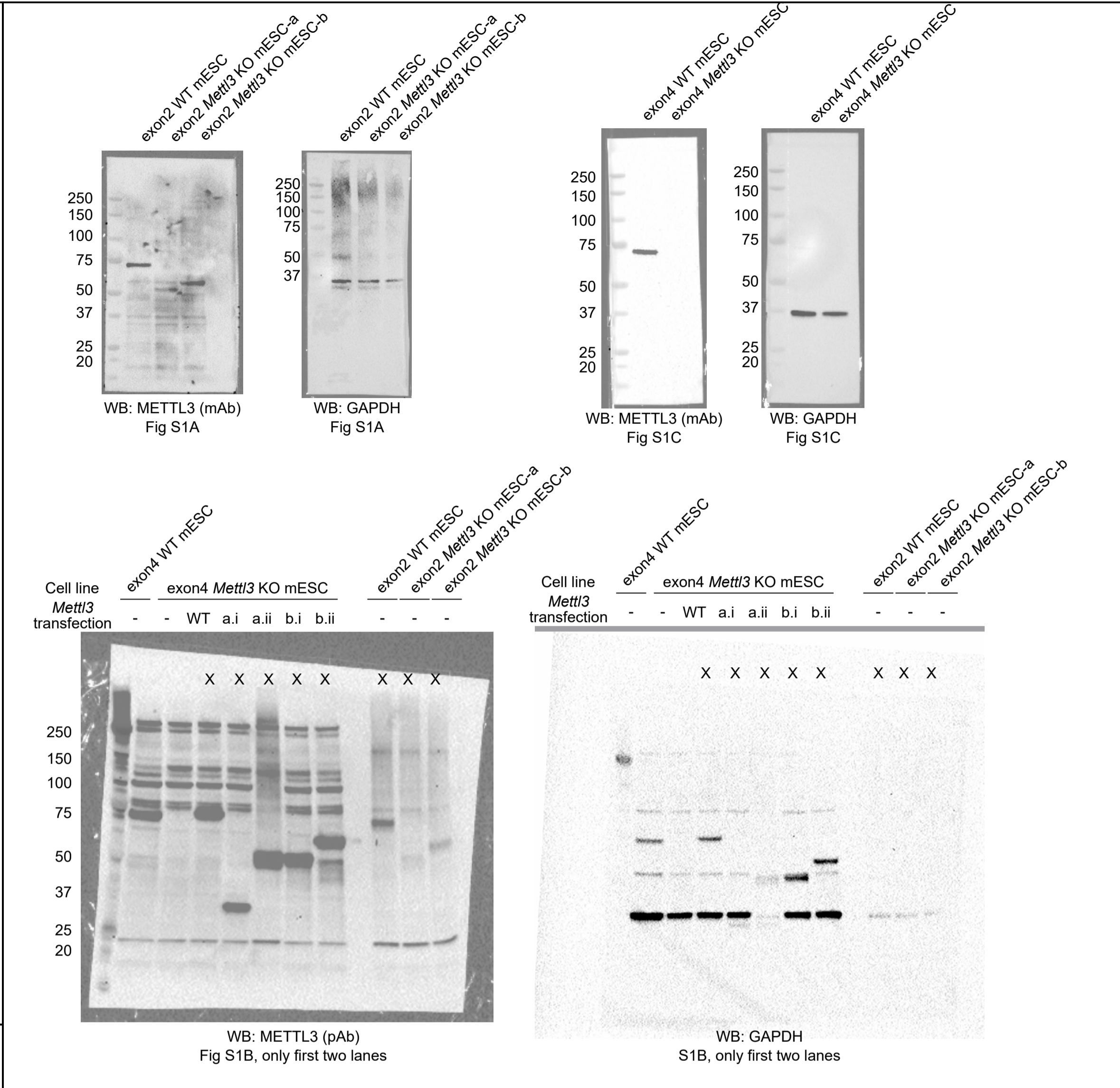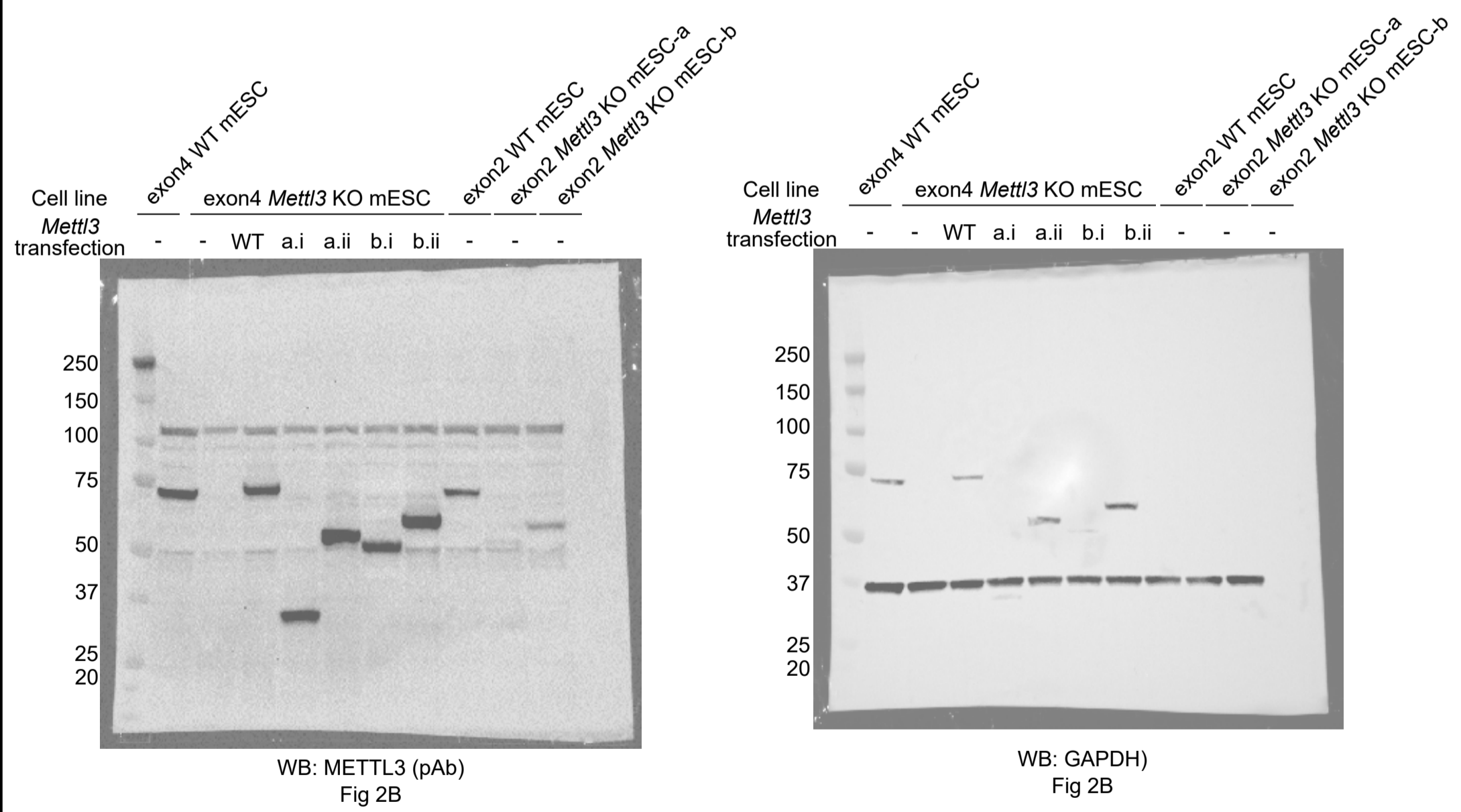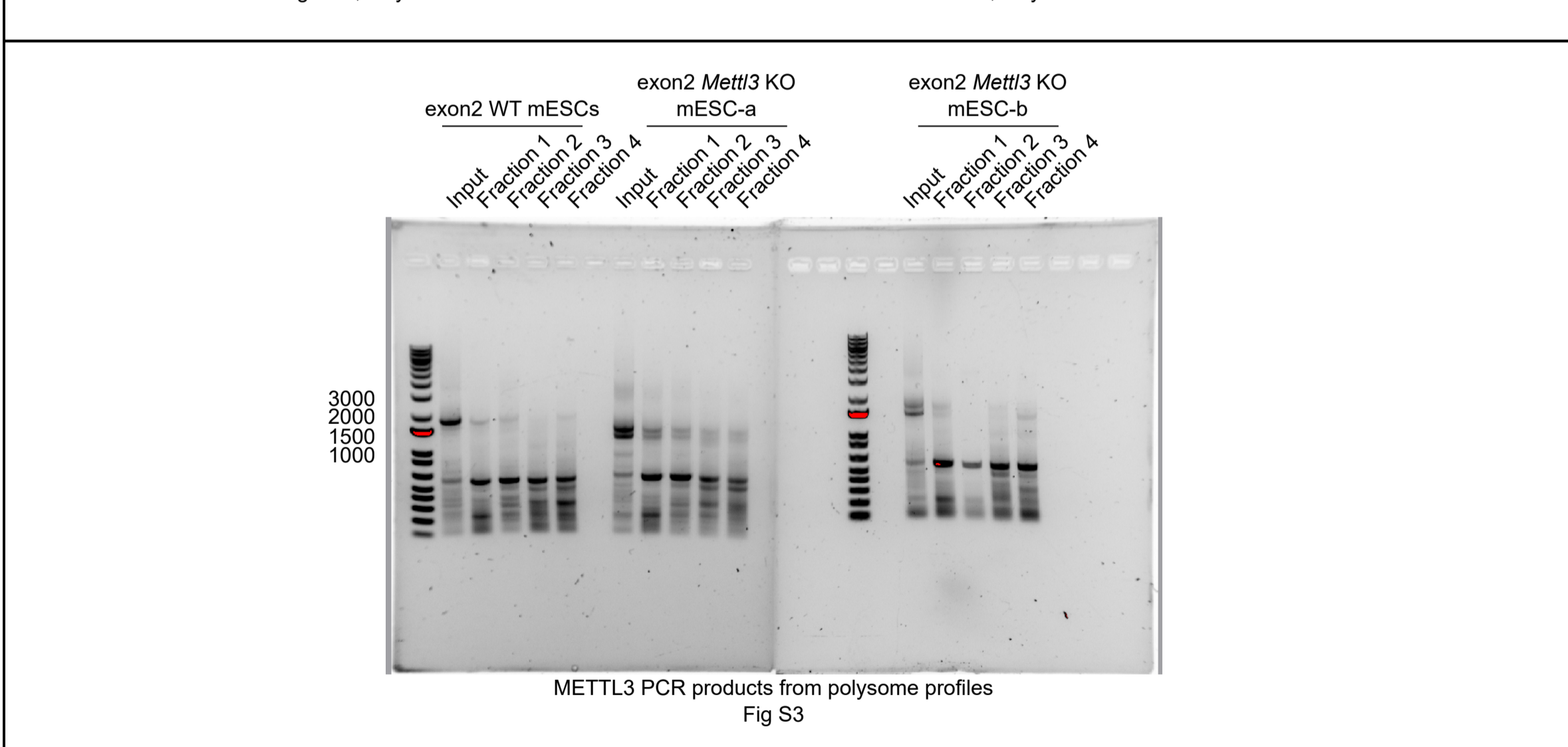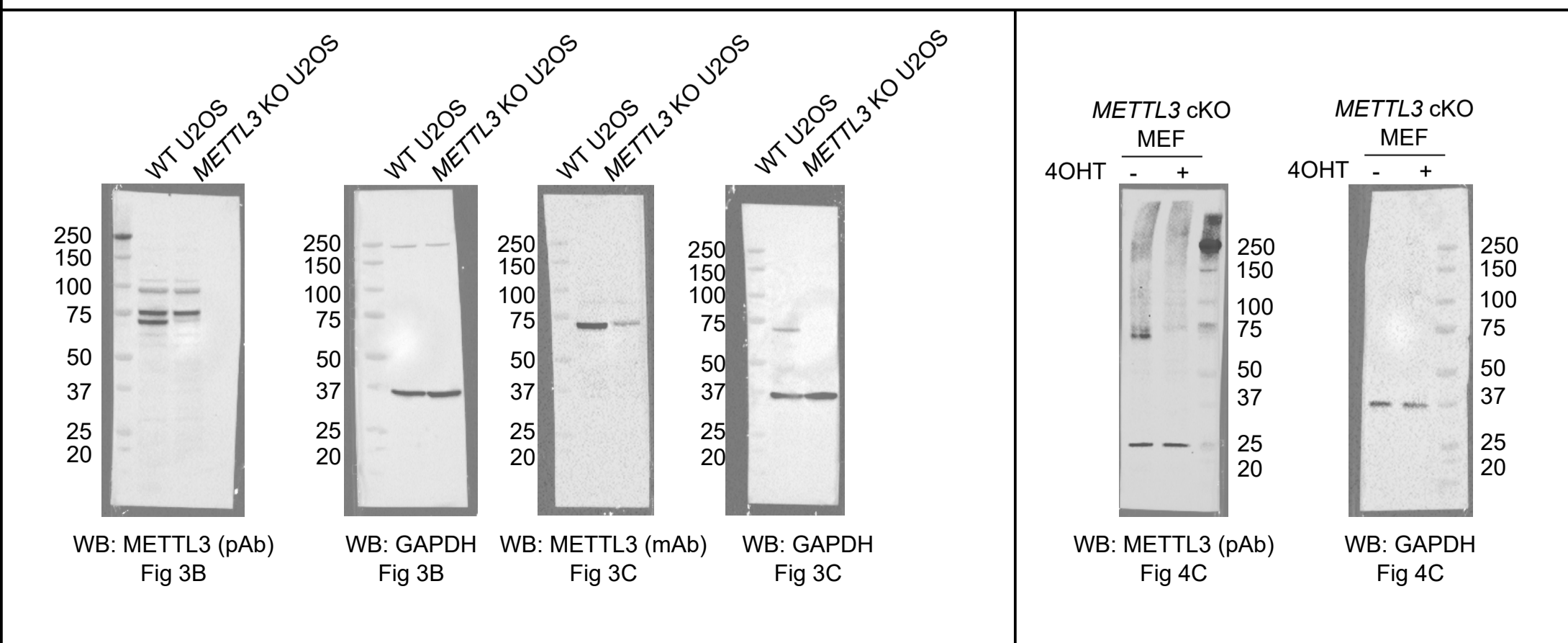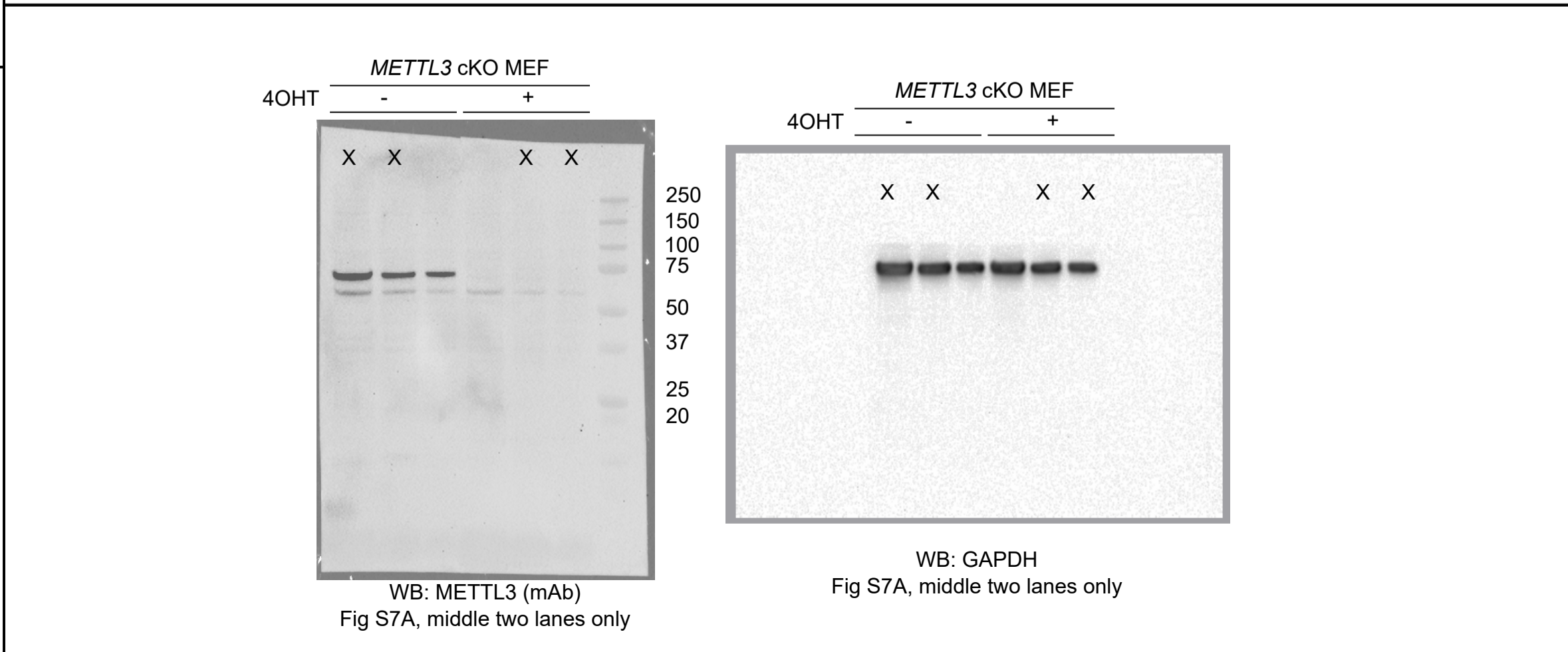

Supplement: S1 Raw images — (PDF) [file pbio.3001683.s014.pdf]
